# Quantification of an Adverse Outcome Pathway Network by Bayesian Regression and Bayesian Network Modeling

**DOI:** 10.1002/ieam.4348

**Published:** 2020-10-23

**Authors:** S Jannicke Moe, Raoul Wolf, Li Xie, Wayne G Landis, Niina Kotamäki, Knut Erik Tollefsen

**Affiliations:** ^1^ Norwegian Institute for Water Research (NIVA) Oslo Norway; ^2^ Norwegian University of Life Sciences (NMBU), Faculty of Environmental Sciences and Natural Resource Management (MINA), Ås Norway; ^3^ Centre for Environmental Radioactivity, Norwegian University of Life Sciences (NMBU), Ås Norway; ^4^ Institute of Environmental Toxicology, Huxley College of the Environment Western Washington University Bellingham Washington USA; ^5^ Finnish Environment Institute (SYKE) Jyväskylä Finland

**Keywords:** Quantitative AOP, Conditional probability tables, Dose–response curve, Key event relationships, Uncertainty

## Abstract

The adverse outcome pathway (AOP) framework has gained international recognition as a systematic approach linking mechanistic processes to toxicity endpoints. Nevertheless, successful implementation into risk assessments is still limited by the lack of quantitative AOP models (qAOPs) and assessment of uncertainties. The few published qAOP models so far are typically based on data‐demanding systems biology models. Here, we propose a less data‐demanding approach for quantification of AOPs and AOP networks, based on regression modeling and Bayesian networks (BNs). We demonstrate this approach with the proposed AOP #245, “Uncoupling of photophosphorylation leading to reduced ATP production associated growth inhibition,” using a small experimental data set from exposure of *Lemna minor* to the pesticide 3,5‐dichlorophenol. The AOP‐BN reflects the network structure of AOP #245 containing 2 molecular initiating events (MIEs), 3 key events (KEs), and 1 adverse outcome (AO). First, for each dose–response and response–response (KE) relationship, we quantify the causal relationship by Bayesian regression modeling. The regression models correspond to dose–response functions commonly applied in ecotoxicology. Secondly, we apply the fitted regression models with associated uncertainty to simulate 10 000 response values along the predictor gradient. Thirdly, we use the simulated values to parameterize the conditional probability tables of the BN model. The quantified AOP‐BN model can be run in several directions: 1) prognostic inference, run forward from the stressor node to predict the AO level; 2) diagnostic inference, run backward from the AO node; and 3) omnidirectionally, run from the intermediate MIEs and/or KEs. Internal validation shows that the AOP‐BN can obtain a high accuracy rate, when run is from intermediate nodes and when a low resolution is acceptable for the AO. Although the performance of this AOP‐BN is limited by the small data set, our study demonstrates a proof‐of‐concept: the combined use of Bayesian regression modeling and Bayesian network modeling for quantifying AOPs. *Integr Environ Assess Manag* 2021;17:147–164. © 2020 The Authors. *Integrated Environmental Assessment and Management* published by Wiley Periodicals LLC on behalf of Society of Environmental Toxicology & Chemistry (SETAC)

## KEY POINTS

- We propose a probabilistic modeling approach for quantification of adverse outcome pathways (AOPs) and AOP networks, based on regression modeling and Bayesian networks.
- We tested this approach with the proposed AOP #245, “Uncoupling of photophosphorylation leading to reduced ATP production associated growth inhibition,” using a small experimental data set from exposure of the plant *Lemna minor* to the pesticide 3,5‐dichlorophenol.
- Each dose–response and response–response (key event) relationship was quantified by Bayesian regression modeling, based on dose–response functions commonly applied in ecotoxicology.
- This proof‐of‐concept study demonstrates that the combined use of regression modeling and Bayesian network modeling is a promising approach for quantification of AOPs, even for data‐poor cases.

## INTRODUCTION

**Figure 1:**
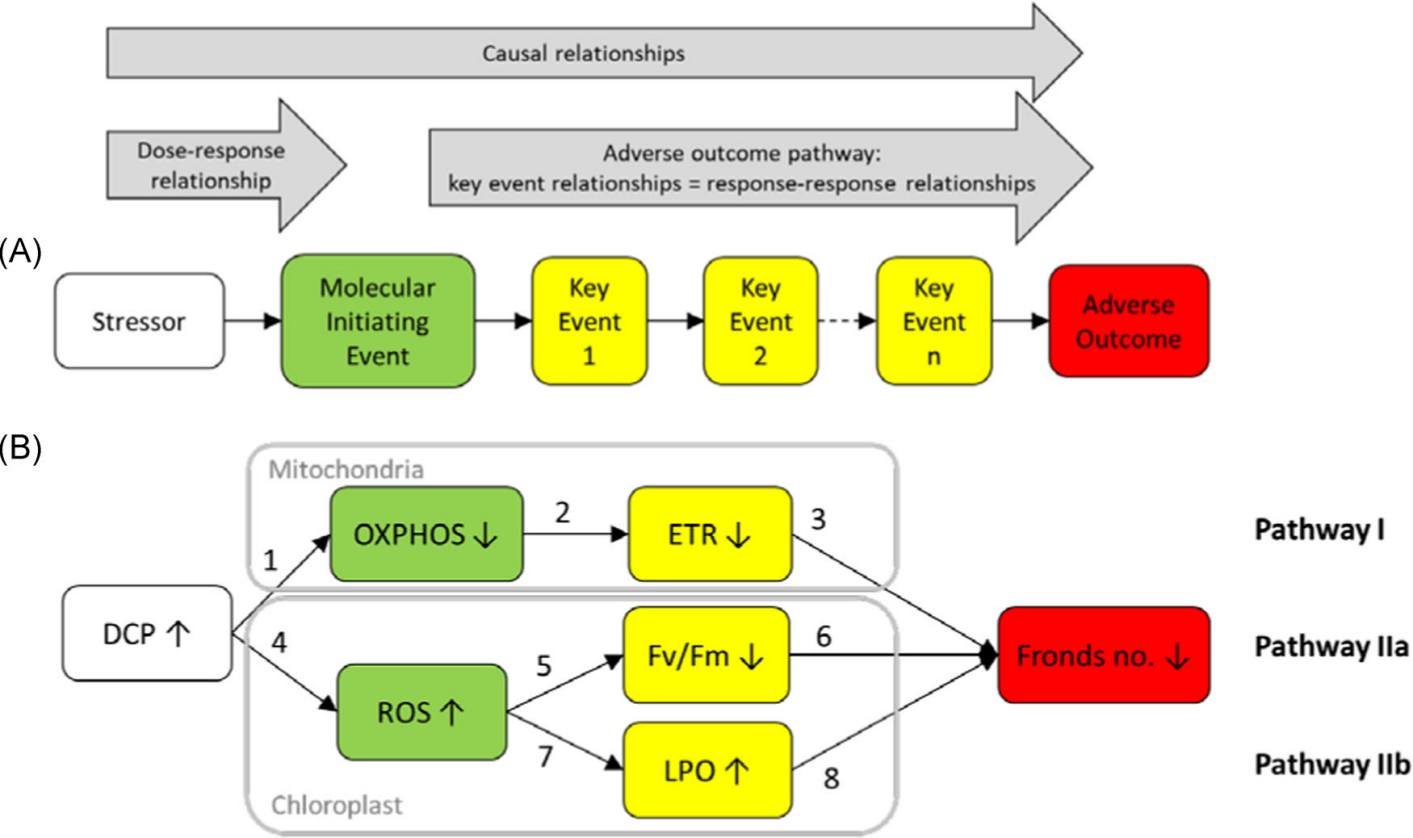
Components of an adverse outcome pathway (AOP) (**A**). Conceptual model of an AOP‐BN (Bayesian network) for quantification of the tentative AOP #245 (see Supplemental Data Figure S1 for more details) (**B**). The nodes are defined in Table 1. The numbered arrows identify the causal relationships defined in Table 2. AOP = adverse outcome pathway; DCP = 3,5‐dichlorophenol; ETR = electron transfer rate; Fv/Fm = maximum quantum yield of photosystem II; LPO = lipid peroxidation; OXPHOS = oxidative phosphorylation; ROS = reactive oxygen species.

The adverse outcome pathway (AOP) framework (Ankley et al. 2010) has gained international recognition as a systematic approach for capturing existing toxicological knowledge to transparently link mechanistic data to apical toxicity endpoints. An AOP model typically describes the causal linkages from a chemical stressor through 3 types of events: 1) a molecular initiating event (MIE) triggered by the stressor, 2) a series of measurable biological responses termed “key events” (KEs), and finally 3) 1 or more adverse outcomes (AOs), which are specialized KEs of regulatory significance (Figure 1A). The KEs are usually ordered by increasing level of biological organization, for example, from cells via tissue to organs, whereas the AO is typically at the level of the individual or even population (Kramer et al. 2011). The causal relationships linking the events are termed “key event relationships” (KERs), and are typically illustrated by arrows in graphical representations of AOPs (Figure 1A).

During the last decade there has been a widespread interest and rapid development in the AOP framework by scientists involved in risk assessment both to human health and to the environment (LaLone et al. 2017). The AOP Knowledge Base (https://aopkb.oecd.org/) combines all available information on AOP development through 4 different information systems. One of these platforms is the AOPWiki (http://aopwiki.org), which holds descriptions of more than 300 proposed AOPs, with status ranging from “under development” to “adopted by the [Organisation for Economic Co‐operation and Development] OECD.” Although the AOPWiki is currently dominated by AOPs relevant for human health, the number of AOPs relevant to other animals and plants is increasing, and the number of taxonomic groups is expanding.

Successful implementation into a regulatory framework is still limited by the lack of quantitative models and assessment of uncertainties associated with AOPs. Although many AOPs may have immediate utility as tools for hazard identification, hypothesis‐driven testing, and prioritization, most are not appropriate for quantitative risk assessment (Conolly et al. 2017). A global horizon scanning exercise, which was recently conducted to address the limitations of the AOP framework in research and regulatory decision making, identified quantification of AOPs as one of the main topical areas (LaLone et al. 2017). Quantitative AOPs (qAOPs) should define the relationships underlying transition from 1 KE to the next sufficiently well to allow quantitative prediction of the probability or severity of the AO occurring for a given activation of the MIE (Conolly et al. 2017). Taking larger advantage of the quantitative nature of these AOPs is considered a key step to further implement the AOP concept into screening, prioritization, and hazard and ultimately risk assessment (Garcia‐Reyero and Murphy 2018).

Although qAOPs can take many forms, a causal modeling framework such as a Bayesian network (BN) (Carriger et al. 2016) seems like a natural choice. A BN is a probabilistic model, usually illustrated as a set of nodes (variables) connected by arrows (causal relationships, alternatively associations) (Figure 1B). The variables are usually discretized into a low number of states, either intervals or mutually exclusive categories. The links are quantified by conditional probability tables (CPTs), which determine the probability distribution of a node (the child node) for all combinations of states of its parent nodes. Bayesian networks therefore allow the propagation of uncertainty quantified throughout the model (Sahlin et al. this issue). Adverse outcome pathways, like BNs, cannot contain feedback loops and are therefore acyclic directed graphs, according to graph theory (Zgheib et al. 2019). More detailed descriptions of BNs are provided by other papers in this special series (e.g., Sahlin et al. this issue). Bayesian networks have been applied extensively in environmental research and management, as reviewed by Kaikkonen et al. (this issue). Many examples can be found in fields such as fisheries management (Uusitalo et al. 2012; Trifonova et al. 2017), water quality assessment (Varis and Kuikka 1997; Borsuk et al. 2004; Moe et al. 2016; Hjerppe et al. 2017), and more recently also in ecological risk assessment and management (Hart and Pollino 2008; Helle et al. 2015; Carriger and Barron 2020; Landis et al. 2020). Nevertheless, BNs have still been used only rarely for the development of quantitative AOPs, beyond a few recent examples (Jaworska et al. 2015; Jeong et al. 2018; Burgoon et al. 2019; Zgheib et al. 2019).

The examples of quantitative AOPs published so far are based mainly on mechanistic models describing systems biology (e.g., Ananthasubramaniam et al. 2015; Miller et al. 2015; Muller et al. 2015; Riedl et al. 2015; Battistoni et al. 2019; Perkins, Ashauer et al. 2019; Perkins, Gayen et al. 2019). A major challenge with this approach is the complexity of the models and the high requirement for data for parametrization. For example, the complete qAOP for renal toxicity based on systems biology has 57 differential equations and 335 parameters (Zgheib et al. 2019). The amount of data required for the calibration of such models is rarely available. Therefore, alternative approaches should be applied that make more efficient use of the available data in combination with other knowledge. In our view, a promising approach is the combined use of BN models to represent the causal structure of an AOP and Bayesian regression models for quantification of exposure–effect relationships and their uncertainty.

An early example of integration of AOP components into a BN can be found in the integrated testing strategy (ITS‐3) for skin sensitization potency assessment (Jaworska et al. 2015), a decision support system for a risk assessor that provides quantitative weight of evidence and formulates an adaptive testing strategy for a chemical. A dynamic BN was developed for quantification of AOP for chronic kidney disease (Zgheib et al. 2019). In a recent example from ecological risk assessment, AOP components were implemented in a BN relative risk model with multiple stressors (Landis et al. 2020). In all these examples, the qAOP has consisted of a single chain with 1 or more KEs organized in a sequence. However, chemical stressors may affect more than 1 MIE or KE, and assessment scenarios typically involve complex mixtures including chemical and nonchemical stressors causing pathway perturbations that interact with multiple MIEs or shared KEs and KERs, that may culminate in 1 or more AOs (Perkins et al. 2011; LaLone et al. 2017). The principles of AOP development within the AOPWiki support construction of AOP networks from simpler units of development (Knapen et al. 2018; Villeneuve et al. 2018).

Bayesian networks are causal, directed, acyclic graphical models (Kjærulff and Madsen 2008), and therefore have optimal properties for implementation of qAOP networks. Nevertheless, to our knowledge there are currently few examples of AOP networks quantified by BN models. These are the AOP network predicting steatosis (abnormal retention of lipids) of human cells (Burgoon et al. 2019) and the AOP network describing the effect of Ag nanoparticles on reproductive toxicity in a nematode (Jeong et al. 2018). In these examples, however, the KEs were defined as semiquantitative nodes, with respectively 2 states (true or false) or 3 states (decrease or stable or increase).

In our study, we used a combination of Bayesian regression analysis and BN modeling to quantify the links and uncertainties of an AOP network, with all nodes discretized into 5 or more intervals. As a case study we have selected the proposed AOP #245, “Uncoupling of photophosphorylation leading to reduced ATP production associated growth inhibition” (https://aopwiki.org/aops/245). This AOP describes the mechanistic linkage between respiratory and photosynthesis inhibition and the adverse effect of growth inhibition in the aquatic macrophyte *Lemna minor* (Xie et al. 2018). In essence, these AOPs describe the causal relationship between chemically induced inhibition of oxidative phosphorylation (OXPHOS) and reduction in the activity of the electron transport chain (ETR), as well as increase in reactive oxygen species (ROS) formation and subsequent inhibition of photosystem II activity (Fv/Fm). Both pathways are assumed to lead to inhibition of growth in *L. minor* (Figure 1B).

In the present paper, we will demonstrate how an AOP—or even an AOP network—can be quantified on the basis of a small experimental data set, by combining observations with expert knowledge and statistical modeling in a Bayesian framework. This relatively simple approach can serve as a proof‐of‐concept for quantification of AOPs and AOP networks based on limited data sets.

## METHODS

### Data

The BN model is based on data from a laboratory experiment (Supplemental Data Table S1) in which the aquatic plant *L. minor* (duckweed) was exposed to the pesticide 3,5‐dichlorophenol (DCP) (Xie et al. 2018). 3,5‐Dichlorophenol belongs to a diverse group of chlorinated phenols, commonly used as pesticides, disinfectants, and as chemical intermediates in the production of more complex chemicals. Chlorophenols cause growth inhibition in primary producers by disrupting energy metabolism, either by uncoupling oxidative or photosynthetic phosphorylation through inhibiting electron transport on inner membrane of mitochondria and thylakoids (Escher et al. 1996).

The measured variables from the experiment included as response variables in the present study are listed in Table 1. These variables were selected based on evidence that they are part of the mechanisms of action for this class of chemicals (Xie et al. 2018). The plant was exposed to DCP in 8 concentrations: 0, 0.5, 1, 1.5, 2, 3, 4, and 8 mg/L. The 2 highest concentrations resulted in death of the plants; therefore, some of the variables could not be measured for these exposure levels (Supplemental Data Table S1). Each response variable was measured in 3 repeated values at each stressor level. This means that replication was not applied at the level of the experimental units, but instead to repeated measurements within each experimental unit. Therefore, the repeated measurements cannot be considered proper replicates. For example, at concentration 0 mg/L, OXPHOS value number 1 is not necessarily connected to ROS value number 1, but equally to ROS values numbers 2 and 3. This is a general problem for response–response relationships when the different response variables are measured from different experimental units. The handling of pseudoreplicates in the statistical modeling is described in the section *Bayesian regression analysis and simulation*.

**Table 1:** Variables from the experimental data set (Supplemental Data Table S1) used as nodes in the AOP‐BN

Node name	Variable name	Description	Unit	Discretization
DCP	3,5‐Dichlorophenol	Chemical stressor; herbicide	mg/L	0; 0.1; 0.22; 0.37; 0.61; 1; 1.65; 2.72; 4.48; 7.39; 12
OXPHOS	Oxidative phosphorylation (inhibition)	Mitochondrial membrane potential	Emitted photons/100 ms	2000; 4000; 6000; 8000; 10 000; 12 000
ROS	Reactive oxygen species	Oxidative stress	Emitted photons/100 ms	2500; 4500; 6500; 8500; 10 500; 12 500
ETR	Electron transfer rate	Reduction in the photosynthetic rate	µmol/(m^2^ s)	0; 6; 12; 18; 24; 30
Fv/Fm	Maximum quantum yield of photosystem II	Reduction in photosynthetic capability	Ratio	0; 0.2; 0.4; 0.6; 1
LPO	Lipid peroxidation	Malondialdehyde content	µg/mol	0; 2; 4; 6; 8; 10
Fronds	Nr of leaves	Individual growth (alternatively, reproduction)	Count	0; 30; 60; 90; 120; 160

AOP = adverse outcome pathway; BN = Bayesian network.

### Structure of the AOP‐BN

The proposed AOP #245 (Xie et al. 2018), which links the mode of action (MoA) of the model respiratory and photosynthesis uncoupler DCP to AOs for *L. minor* *,* is illustrated in Supplemental Data Figure S1. The present study suggests that DCP displays both concentration‐dependent and target‐specific MoAs that seem to be causally related. When exposed to plants, DCP can disrupt energy transduction by uncoupling oxidative and photosynthetic phosphorylation through inhibiting the electron transport in the inner membrane of mitochondria and thylakoids (Escher et al. 1996). Additionally, the phenolic compounds have also been proposed to inhibit electron transport in the chloroplast through disruption of the photosystem I and disrupt photoreduction of NADP^+^ (Ohad and Hirschberg 1992; Plekhanov and Chemeris 2008). Some responses, including reduction in OXPHOS, ETR, and fronds number, were observed at low DCP concentration (0.5–1 mg/L), which indicates that these endpoints were directly associated with the respiratory and photosynthesis uncoupling activity of DCP. Effects observed at higher concentrations (>1 mg/L), such as ROS formation, lipid peroxidation (LPO), and modulation of PSII efficiency, indicated that these endpoints were associated with excessive ROS formation and oxidative damage to key cellular components in *L. minor*. Thus, the proposed network of AOPs describes how low stressor levels can trigger the first MIE (OXPHOS) in mitochondria (and representing 1 toxicity pathway), while higher stressor levels can further trigger the second MIE (ROS production) in chloroplasts (i.e., representing another toxicity pathway), given that higher stressor levels also trigger ROS (Supplemental Data Figure S1).

The AOP‐BN model was implemented in the software Netica version 6.04 (Norsys Software Corp., Vancouver, Canada; http://www.norsys.com). The qualitative structure of the AOP‐BN is a simplified version of the proposed AOP (Figure 1B), including only the KEs for which measured responses were available from the experiment (Xie et al. 2018). The AOP‐BN is a network consisting of 3 chains (i.e., pathways) with the same chemical stressor (DCP) and AO (reduced fronds number). Both OXPHOS and ROS are defined as MIEs, while the 3 variables ETR, Fv/Fm, and LPO are all defined as KEs. An AOP by definition starts with an MIE and should therefore be chemical agnostic (Ankley et al. 2010). In our study, however, a stressor gradient is needed to evaluate the performance of the quantified AOP‐BN. We have therefore included the stressor as the parent node in the AOP‐BN, although the BN could also have been constructed with the 2 MIEs as parent nodes.

The nodes of a BN are typically defined by a limited number (3–10) of discrete states, which can be categorical or intervals. In principle it is also possible to include continuous variables (Qian and Miltner 2015; Moe et al. 2020). However, BN software packages that allow continuous variables have requirements such as normal distribution (Kaikkonen et al. this issue), which was not appropriate for the variables in our study (Table 2). For example, assuming a normal distribution might result in negative values for some of the counts data. For this AOP‐BN, all variables were discretized into 5 intervals, except for the stressor node, which had 10 intervals. The high number of stressor intervals was chosen to make the BN more sensitive to changes along the stressor gradient. Dose–response relationships typically show a sigmoid response curve with a steep increase at some intermediate dose range, which was expected to occur at different ranges of the stressor gradient for Pathway I versus Pathways IIa and IIb. For all other nodes, to obtain a simple model structure and interpretation, we limited the discretization to 5 intervals. A comparable number of states, 3 to 6 intervals, were used in the BN models by Jaworska et al. (2015) and by Landis et al. (2020). A higher number of states would still be feasible with this approach because the quantification of CPTs is based on a high number of simulated values. The discretization was defined by equidistant intervals within the range of each node (Table 1). The DCP node had equidistant intervals at logarithmic scale, given that the predictor variable is often log‐transformed in dose–response models. For each node, the range was set from approximately 67% of the minimum observed value to 150% of the maximum observed value.

**Table 2:** Regression models for causal relationships^a^

Relationship nr	Predictor variable	Response variable	Response variable type	Response variable distribution	Response function (*drc*)	Goodness of fit (*R* ^2^)
1	DCP	OXPHOS	Count	Poisson	LL.5	0.98
2	OXPHOS	ETR	Continuous, positive	Truncated Gaussian^a^	LL.4	0.89
3	ETR	Fronds	Count	Poisson	AR.3	0.96
4	DCP	ROS	Count	Poisson	LL.5	0.79
5	ROS	Fv/Fm	Ratio of 2 counts	Binomial	LL.4	0.65
6	Fv/Fm	Fronds	Count	Poisson	AR.3	0.97
7	ROS	LPO	Continuous, positive	Truncated Gaussian^b^	LL.4	0.68
8	LPO	Fronds	Count	Poisson	LL.4	0.52

DCP = 3,5‐dichlorophenol; ETR = electron transfer rate; Fv/Fm = maximum quantum yield of photosystem II; LPO = lipid peroxidation; OXPHOS = oxidative phosphorylation; ROS = reactive oxygen species.

^a^ Response functions described for the R package *drc* (Ritz et al. 2015): LL.4 = log‐logistic with 4 parameters; LL.5 = log‐logistic with 5 parameters; AR.3 = shifted asymptotic regression (lower limit shifted from 0). For more information on the variables, see Table 1.

^b^ Continuous distributions were truncated with a lower bound of 0 to prevent simulation of negative values for ETR and LPO.

The causal relationships in a BN model are typically displayed by arrows, which are technically termed “edges” or “arcs.” In Figure 1B, only the node‐connecting arrows numbered 1 and 4 (DCP → OXPHOS and DCP → ROS) represent true dose–response relationships, whereas the remaining numbered arrows represent KERs, which are also referred to as “response–response relationships” (Doering et al. 2019). For simplicity, in the present paper, we will refer to both dose–response and response–response relationships as causal relationships. The arrows of a BN are quantified by CPTs, which determine the probability distribution of a child node conditionally on the probability distribution of the parent nodes. The values of a CPT thus correspond to parameters in process‐based models. In our study, the CPTs were quantified by statistical modeling. However, CPTs can also be quantified by other methods, for example, by frequency distributions (counts) of observations, by theory, or by expert judgment (for examples, see Moe et al. 2020).

A limitation of traditional BN models is that they cannot contain feedback loops, which are essential parts of biological systems. Feedback systems can occur at all levels of biological organization, from homeostatic mechanisms operating at the level of cells, tissues, or physiological processes, to density‐dependent regulation occurring at the population level. More advanced dynamic BN models, which have been developed only recently, can incorporate feedback loops explicitly (Zgheib et al. 2019). Here we have applied a more common approach: to let the modeled relationship represent a steady‐state situation for a given timeframe, with any feedback incorporated implicitly.

### Bayesian regression analysis and simulation

In the present study, all CPTs were quantified by empirical modeling of the relationships. The number of observations (maximum 24 observations per relationship) was too low to allow for quantification of CPTs based directly on the counts. The counts would have been insufficient even if the number of states per node were reduced to 3, resulting in CPTs with 3 × 3 = 9 values. A statistical modeling approach can exploit the limited number of observations more efficiently by combining expert knowledge or assumptions regarding the structure of the causal relationship with the patterns and the variability in the data.

**Figure 2:**
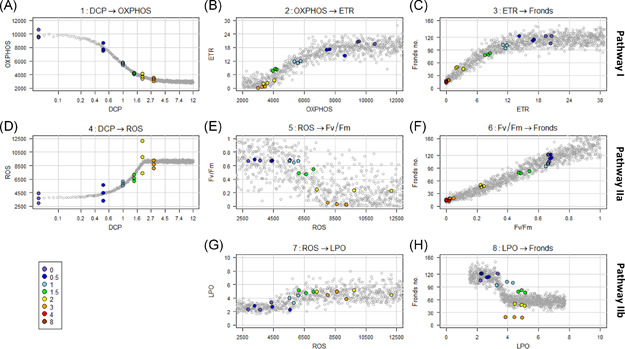
Observed and simulated causal relationships: dose–response (**A**, **D**) and response–response (**B**, **C**, **E**–**H**) relationships. Colored dots show the measured values; the color code indicates the experimental treatment dose (see legend) in mg/L. The grey dots are simulated response values; a subset of 1000 out of 10 000 simulated values is displayed. For plots (**A**) and (**D**), the *x*‐axis is in log‐scale. The values DCP = 0 mg/L are displayed at 0.05 mg/L. The vertical and horizontal grid lines correspond to the intervals of the BN nodes (Table 1). The regression models are described in Table 2. AOP = adverse outcome pathway; BN = Bayesian network; DCP = 3,5‐dichlorophenol; ETR = electron transfer rate; Fv/Fm = maximum quantum yield of photosystem II; LPO = lipid peroxidation; OXPHOS = oxidative phosphorylation; ROS = reactive oxygen species.

**Figure 3:**
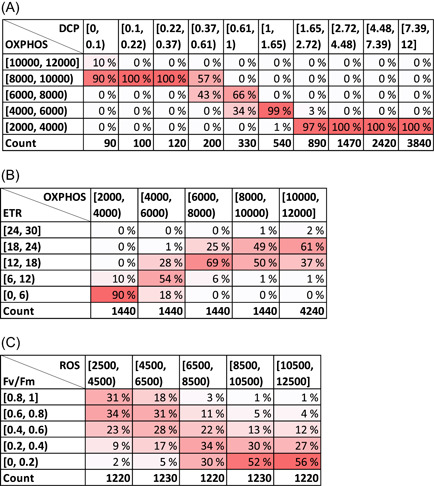
Examples of CPTs generated from simulated data, illustrating causal relationships with different levels of variability. Low variability: dose–response relationship DCP → OXPHOS (Figure 2A) (**A**). Intermediate variability: key event relationship OXPHOS → ETR (Figure 2B) (**B**). High variability: key event relationship ROS → Fv/Fm (Figure 2E) (**C**). The remaining CPTs are shown in Table S2. The CPTs shown here are transposed for alignment with the plots in Figure 2. CPT = conditional probability tables; DCP = 3,5‐dichlorophenol; ETR = electron transfer rate; Fv/Fm = maximum quantum yield of photosystem II; OXPHOS = oxidative phosphorylation; ROS = reactive oxygen species.

Our approach to quantifying the CPTs had 4 main steps. For each causal relationship,

1) select an appropriate regression model based on explorative data analysis, model selection criteria, and/or expert knowledge of the MoA (Table 2).
2) fit the regression model to the data to obtain parameter estimates (with posterior probability distributions), which can generate dose– or response–response curves (Supplemental Data Figure S2).
3) use the fitted regression model to simulate a large number of response values along a gradient of predictor values, reflecting the uncertainty in the causal relationship (Figure 2).
4) calculate the probability distributions for the CPT as the frequency distribution of the simulated values (Figure 3).

Dose–response modeling is the state‐of‐the‐art methodology underlying modern risk assessment, with the log‐logistic function as one of the most commonly used models (Ritz 2010). In the present study, we chose to apply a Bayesian regression model to quantify the relationships. The differences between Bayesian statistics and the more common frequentist or classical statistical modeling have been described elsewhere (Kruschke et al. 2012; Baldwin and Larson 2017; Sahlin et al. this issue). In brief, a frequentist linear regression model will provide a parameter estimate for the slope (and for the intercept) as a point value with an interval (standard error). In contrast, a Bayesian linear regression model will provide a predefined number of simulated values for the slope (and for the intercept), from which a probability distribution can be defined, as well as a point value with standard error if desirable. In simple cases like a linear regression, the frequentist and Bayesian methods will typically provide very similar results. However, the Bayesian method can in addition provide full probability distributions for the fitted parameters, which was convenient for quantifying the CPTs.

The functional form of regression models for the AOP‐BN were based on the set of dose–response curve models described by Ritz et al. (2015), and implemented in the package *drc* of the open‐source statistical software R (R Core Team 2020). Here, we assumed that the dose–response functions most commonly used in ecotoxicology would also be suitable for characterizing the KERs of the AOP‐BN. Initial explorative analyses of the causal relationships were carried out with frequentist regression in *drc*, to take advantage of the built‐in functionality for model specification and selection. This explorative data analysis guided our selection of dose–response function for each relationship, which we subsequently implemented in a Bayesian regression framework (Table 2).

All relationships (Table 2) were fitted to the data using the R package *brms* (Bürkner 2017), which is a high‐level interface to statistical inference language Stan (Carpenter et al. 2017). This package allows Bayesian generalized nonlinear multivariate multilevel models with full Bayesian inference, supporting a wide range of distributions. For each response variable, we selected a suitable distribution such as Poisson for counts and binomial for ratios, with a corresponding link function (Table 2). In this Bayesian regression package (Bürkner 2017), specification of prior distributions for parameters is flexible and encourages users to apply prior distributions that reflect their beliefs. Weakly informative priors were chosen for all parameters in our models. In the Markov Chain Monte Carlo (MCMC) resampling, 2000 samples were drawn from 4 independent chains, with the first half of the samples being used for warmup, resulting in 4000 posterior samples for each model. All models converged satisfactorily with the convergence diagnostic $\hat{R}$ values ≤1.01. The frequentist regression models initially fitted with the *drc* package could in principle also be used to simulate new response values, based on the estimated parameters and standard error. However, our initial efforts with this simpler approach resulted in simulated values that were not meaningful, such as negative concentrations.

The fitted Bayesian regression models (Table 2) were subsequently used for simulating a high number of response values. For each model, the set of 3 to 5 parameters was estimated as joint posterior probability distributions by their respective Bayesian regression models. Each parameter set drawn from this posterior distribution corresponds to a predicted dose–response (or response–response) curve, as illustrated by the 10 randomly drawn curves in Supplemental Data Figure S2. In our approach, for every causal relationship (Table 2), 10 000 data points were simulated from the joint posterior distributions of parameters. For each predictor variable, a vector of artificial predictor values was created, spanning from 67% to 150% of the reported range of this variable, with a total vector length of 1000 equally spaced values. For each of these predictor values, 10 parameter sets were drawn from the joint posterior distribution, resulting in 10 simulated response values. The resulting simulated 10 000 response values were subsequently used for the construction of the CPTs (Supplemental Data Table S2).

### Quantification of the AOP‐BN

The CPTs were parameterized by counting the simulated values falling into each combination of parent and child node states. For example, consider the relationship DCP → OXPHOS (Figure 3A). For the first state of the parent node DCP (Figure 3A column [0, 0.1]), we let the probability distribution of the child node OXPHOS correspond to the frequency distribution across the grid cells (percentages: 10%, 90%, 0%, 0%, 0%). The probability distributions of the CPT illustrated in Figure 3A thereby reflect variation in the simulated causal relationship in Figure 2A. (Note that the tables in Figure 3 are arranged to reflect the plots in Figure 2; CPTs may have different layouts in different BN software packages).

For the simulation of causal relationships, the range of the *x*‐axis was defined by an extension of the observed range of values from 67% to 150% (described in the section *Bayesian regression analysis and simulation*). The purpose of extending the range in both directions was to ensure a sufficient range of *y*‐values for each relationship. For the quantified AOP‐BN model, however, we selected a narrower range for some of the variables, for example, after an asymptotic response value was reached. In consequence, the count of simulated values on which the CPTs are based can differ among the relationships (see Figure 3B vs Figure 3C), but the counts were always sufficiently high to produce reasonable distributions.

The AO node—fronds number—was modeled as a response variable to each of the 3 KEs separately (Figures 2C, 2F, and 2H). Hence, the 3 resulting CPTs needed to be integrated in some way. We tested 4 alternative combination rules with different weighting of the 3 pathways (Table 3). Rule 1 assumes equal weight of the 3 pathways, whereas Rules 2 and 3 assume lower weight of Pathways IIa and IIb, which are initiated at higher stressor levels than Pathway I. Rule 4 assumed all weight of Pathway I, which was considered representative for the low stressor level. The performance of the AOP‐BN was evaluated for all 4 alternative combination rules (see the section *Model evaluation* and Supplemental Data Figure S3).

**Table 3:** Three alternative rules for combination of the 3 pathways (see Supplemental Data Figure S3), by differential weighting of the 3 pathways

Pathway	Nodes	Weights in combination of pathways			
		Rule 1	Rule 2	Rule 3	Rule 4
I	OXPHOS → ETR → Fronds number	33%	50%	75%	100%
IIa	ROS → Fv/Fm → Fronds number	33%	25%	12.5%	0%
IIb	ROS → LPO → Fronds number	33%	25%	12.5%	0%

ETR = electron transfer rate; Fv/Fm = maximum quantum yield of photosystem II; LPO = lipid peroxidation; OXPHOS = oxidative phosphorylation; ROS = reactive oxygen species.

Although our approach to quantification of the AOP‐BN was based on statistical modeling, expert judgment was applied in different steps of this procedure; in particular, use of more automated processes was constrained by lack of data. In summary, expert judgment was applied as follows:

1) Selection of variables (Table 1): All variables with measured responses belonging to AOP #245 were included, even if observations were missing for some of the treatment levels.
2) Model selection (Table 2): The choice of regression model for each causal relationship, including the distribution, priors, and other constraints, was based on visual inspection of the data and understanding of the underlying data‐generating processes. This judgment was supplemented by the model selection functionality in the R package *drc*, and evaluated by test statistics (*R* ^2^).
3) Discretization of variables (Table 1): Practical considerations were applied in setting the range of each variable (based on observed and simulated values), the number of intervals (limited to 5 for all child nodes), and the size of the intervals (equidistant).
4) Integration of the 3 pathways (Table 3): Best guesses were applied in assigning weights to the pathways for the 4 alternative combination rules.

### Model evaluation

The sensitivity of the AO node (fronds number) to each of the parent nodes in the AOP‐BN was analyzed by the built‐in function “Sensitivity to findings” in Netica. In environmental modeling, sensitivity analysis can help to determine which parts of the model affect the variables of interest the most, thereby identifying which parts should be made with caution and a high level of accuracy. The sensitivity is measured as mutual information between the target node and the parent node, which corresponds to the reduction in entropy of the target node (measured in bits) due to a finding (evidence) at the parent node (Table 3).

The performance of the AOP‐BN was evaluated by internal validation, that is, by using the same data source that was used for quantifying the model (Sahlin et al. this issue). External validation using a separate data set would be preferable, but our data set (Supplemental Data Table S1) was too small for splitting into a training and a testing set, and a suitable independent data set was not available. In our case, the accuracy of the model predictions was evaluated by comparing the predicted AO state at a given stressor level with the observed AO state at the same stressor level (Table 4, Supplemental Data Figure S4). This test was carried out with the AOP‐BN instantiated from either the stressor node, or the MIE nodes or the KE nodes. Furthermore, the accuracy was evaluated for 3 alternative levels of resolution of the AO node: 5, 3, or 2 states (Table 5). For the 3‐states resolution, the 3 lowest intervals (0–90 fronds) of the original 5 states were merged. For the 2‐states resolution, the 2 upper intervals were also merged (90–160 fonds).

**Table 4:** Sensitivity analysis for the AOP‐BN with the 3 alternative combination rules for the 3 pathways (Table 3)^a^

Node type	Node	Mutual information			
		Rule 1	Rule 2	Rule 3	Rule 4
Stressor	DCP	30.2%	32.8%	17.8%	30.3%
MIE	OXPHOS	29.8%	33.3%	17.1%	32.8%
MIE	ROS	31.5%	32.9%	19.4%	28.0%
KE	ETR	29.2%	35.5%	15.4%	43.4%
KE	Fv/Fm	30.1%	24.2%	11.7%	11.5%
KE	LPO	21.2%	19.1%	29.3%	12.0%

AOP = adverse outcome pathway; BN = Bayesian network; DCP = 3,5‐dichlorophenol; ETR = electron transfer rate; Fv/Fm = maximum quantum yield of photosystem II; KE = key event; LPO = lipid peroxidation; MIE = molecular initiating event; OXPHOS = oxidative phosphorylation; ROS =reactive oxygen species.

^a^ The values show the percentage of mutual information between the target node “Fronds number” and the parent nodes. Higher values represent higher sensitivity of the target node to the parent node.

**Table 5:** Accuracy rates of the predicted AO (reduction in fronds number) by the AOP‐BN model, using the 4 alternative combination rules (Table 3) and 3 alternative resolutions of the AO node (5, 3, or 2 states)^a^

Resolution of the AO node	Combination rule (% weighting)	Accuracy		
		From stressor	From MIE	From KE
5 states	Rule 1 (33; 33; 33)	37.5%	61.1%	61.1%
	Rule 2 (50; 25; 25)	37.5%	61.1%	61.1%
	Rule 3 (75; 12.5; 12.5)	37.5%	61.1%	61.1%
	Rule 4 (100; 0; 0)	87.5%	77.8%	77.8%
3 states	Rule 1 (33; 33; 33)	62.5%	77.8%	83.3%
	Rule 2 (50; 25; 25)	62.5%	77.8%	83.3%
	Rule 3 (75; 12.5; 12.5)	62.5%	83.3%	83.3%
	Rule 4 (100; 0; 0)	100%	88.9%	88.9%
2 states	Rule 1 (33; 33; 33)	100%	94.4%	100%
	Rule 2 (50; 25; 25)	100%	94.4%	100%
	Rule 3 (75; 12.5; 12.5)	100%	100%	94.4%
	Rule 4 (100; 0; 0)	100%	88.9%	88.9%

AO = adverse outcome; BN = Bayesian network; KE = key event; MIE = molecular initiating event.

**Figure 4:**
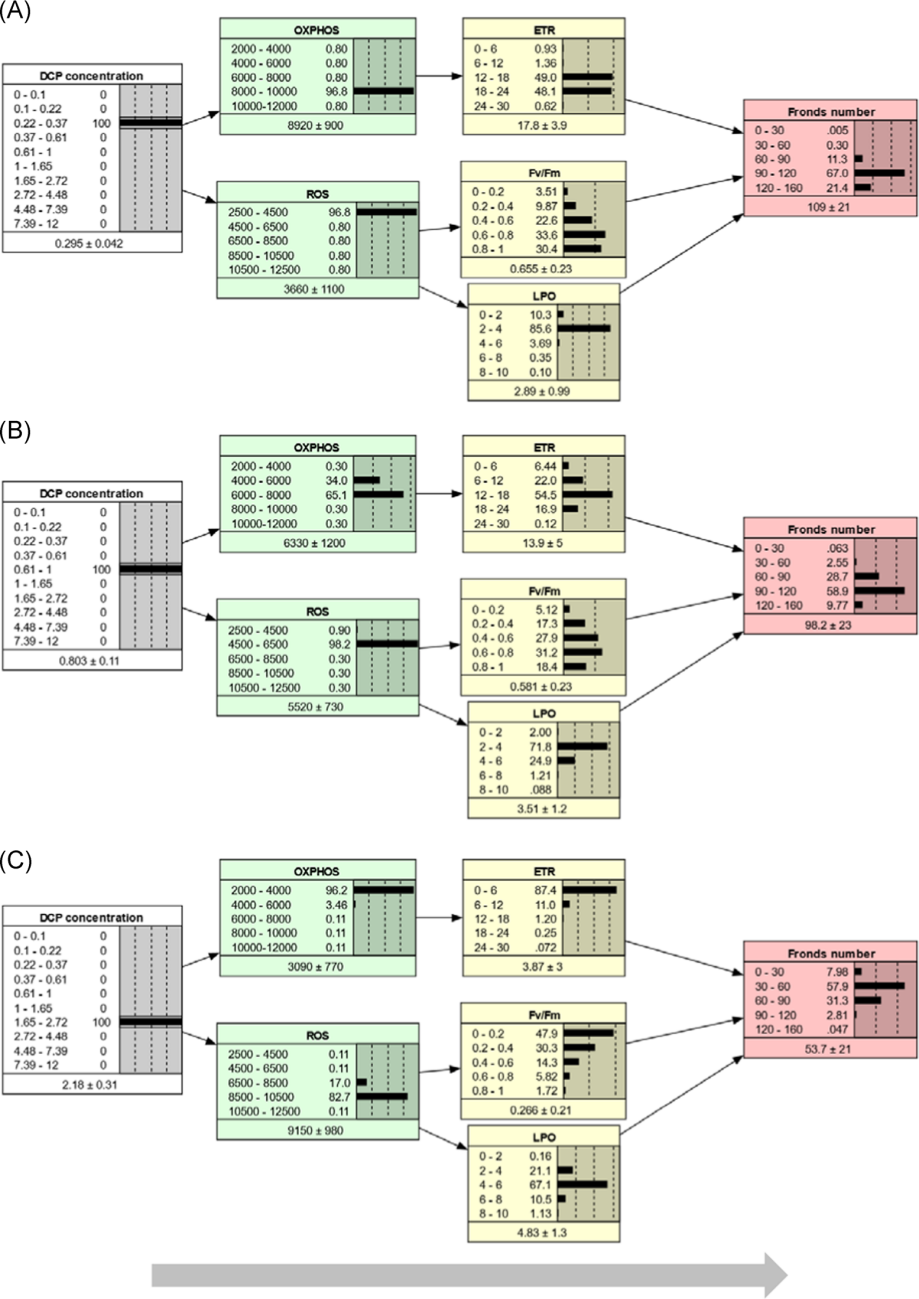
Prognostic inference. The AOP‐BN model is run forwards from the stressor node with evidence set at different stressor concentration intervals: low (**A**), intermediate (**B**), high (**C**). In each node, the states (intervals) are shown to the left, while the probabilities are shown to the right both as values and as bars. The mean and standard deviation are given below each node. Evidence entered to a node is indicated by thin lines around the bar with 100% probability. AOP = adverse outcome pathway; BN = Bayesian network; DCP = 3,5‐dichlorophenol; ETR = electron transfer rate; Fv/Fm = maximum quantum yield of photosystem II; LPO = lipid peroxidation; OXPHOS = oxidative phosphorylation; ROS = reactive oxygen species.

^a^ The AOP‐BN is instantiated with evidence (Supplemental Data Table S1) for either the stressor node, the MIE nodes, or the KE nodes (cf. Figures 4 and 6). For more details, see the section *Evaluation*.

## RESULTS AND DISCUSSION

### Quantification of the AOP‐BN model

The quantified dose–response relationships and KERs are displayed in Figure 2. The expected direction of a relationship is given by the combination of the up–down arrows in the 2 connected nodes of the conceptual model (Figure 1B): Two nodes with up–down arrows pointing in the same direction represent a positive correlation. All estimated relationships (Figure 2) displayed the expected direction according to the conceptual model (Figure 1). For example, relationship number 5 consists of 2 nodes with up–down arrows pointing in opposite directions (increasing ROS and decreasing Fv/Fm), which is consistent with the negative relationship in Figure 2E. Conversely, relationship number 6 has 2 nodes with up–down arrows both pointing in the same direction (decreasing Fv/Fm and decreasing fronds number), which is consistent with the positive relationship in Figure 2F.

The goodness‐of‐fit of each fitted function is measured by the coefficient of determination, *R* ^2^ (Table 2). For most of the relationships, the displayed variation in simulated values corresponds well with the variation of the measured values. Nevertheless, there are cases in which the selected model seems to overfit (Figure 2A, *R* ^2^ = 0.98) or underfit (Figure 2E, *R* ^2^ = 0.65) to the data. These tendencies will be described in more detail in the section *AOP‐BN model prediction and evaluation*.

The 2 stressor–MIE relationships, DCP → OXPHOS (Figure 2A) and DCP → ROS (Figure 2D), displayed the least variability of simulated values. These were both fitted with a 5‐parameter log‐logistic function with Poisson distribution. For DCP → OXPHOS, the low variation of the simulations is reflected in the sharp distributions of the corresponding CPT (Figure 3A). In the case of DCP → ROS, we would expect a higher variability of simulated values, to represent the variation of the measured values (*R* ^2^ = 0.79). This suggests that the selected regression model may not be able to fully represent the variability when applied for simulation. This was also the case with the KER of LPO → Fronds (Figure 2H), which had the lowest *R* ^2^ (0.52).

The fitted KER displaying the highest variability was ROS → Fv/Fm (Figure 2E), with *R* ^2^ = 0.65. The relatively poor fit is reflected in the wide probability distributions in the CPT (Figure 3C). Because the response variable Fv/Fm is a ratio of 2 counts, a binomial distribution was used for the variable. Since the *y*‐axis has the range 0 to 1, the deviation in absolute values is far lower than for most of the other variables (e.g., LPO, with the range 0–10). Two other distributions, beta and lognormal, were tested for the variable Fv/Fm and found to result in overfitting or otherwise not representing the variation in the measured values. Following this expert judgment, we chose the binomial distribution despite the high variation of simulated values.

The remaining relationships (Figures 2B, 2C, 2F, and 2G) all had relatively high *R* ^2^ values in the range 0.68 to 0.96. These KERs were parameterized by 2 types of functions (Table 2): 4‐parameter log‐logistic with truncated Gaussian distribution or shifted asymptotic regression with Poisson distribution. Further testing of alternative model parametrizations might further improve the goodness of fit for each relationship, at least if a larger data set becomes available. For now, we consider the selected models to be suitable for the purpose of our AOP‐BN modeling exercise. All CPTs resulting from these quantified relationships are available in Supplemental Data Tables S2a to S2i. The large variation in fitted regression curves (Supplemental Data Figure S2), goodness‐of‐fit scores (Table 2), and patterns of simulated values (Figure 2) underlines the importance of carefully selecting and adapting a regression model to each individual KER for the generation of representative CPTs. A more straightforward approach could be to implement a fitted dose–response equation directly in the BN software. However, CPTs based directly on equations would not be able to represent the specific patterns of variability in each of the causal relationships, in as detailed a manner as the CPTs based on our combined estimation and simulation approach (Figure 3).

The quantification of this AOP‐BN network must be completed by integrating the 3 pathways into the 1 AO node (Figure 1A). For this purpose, we constructed the model with 4 alternative combination rules, assigning different weights to Pathway I relative to Pathways IIa and IIb (Table 3). Combination Rule 4 (100% weight to Pathway I) corresponds to a single linear AOP. The performance of the AOP‐BN was evaluated by running the model from the stressor, from the MIEs, and from the KERs, respectively (Table 5; see next section).

### AOP‐BN model prediction and evaluation

**Figure 5:**
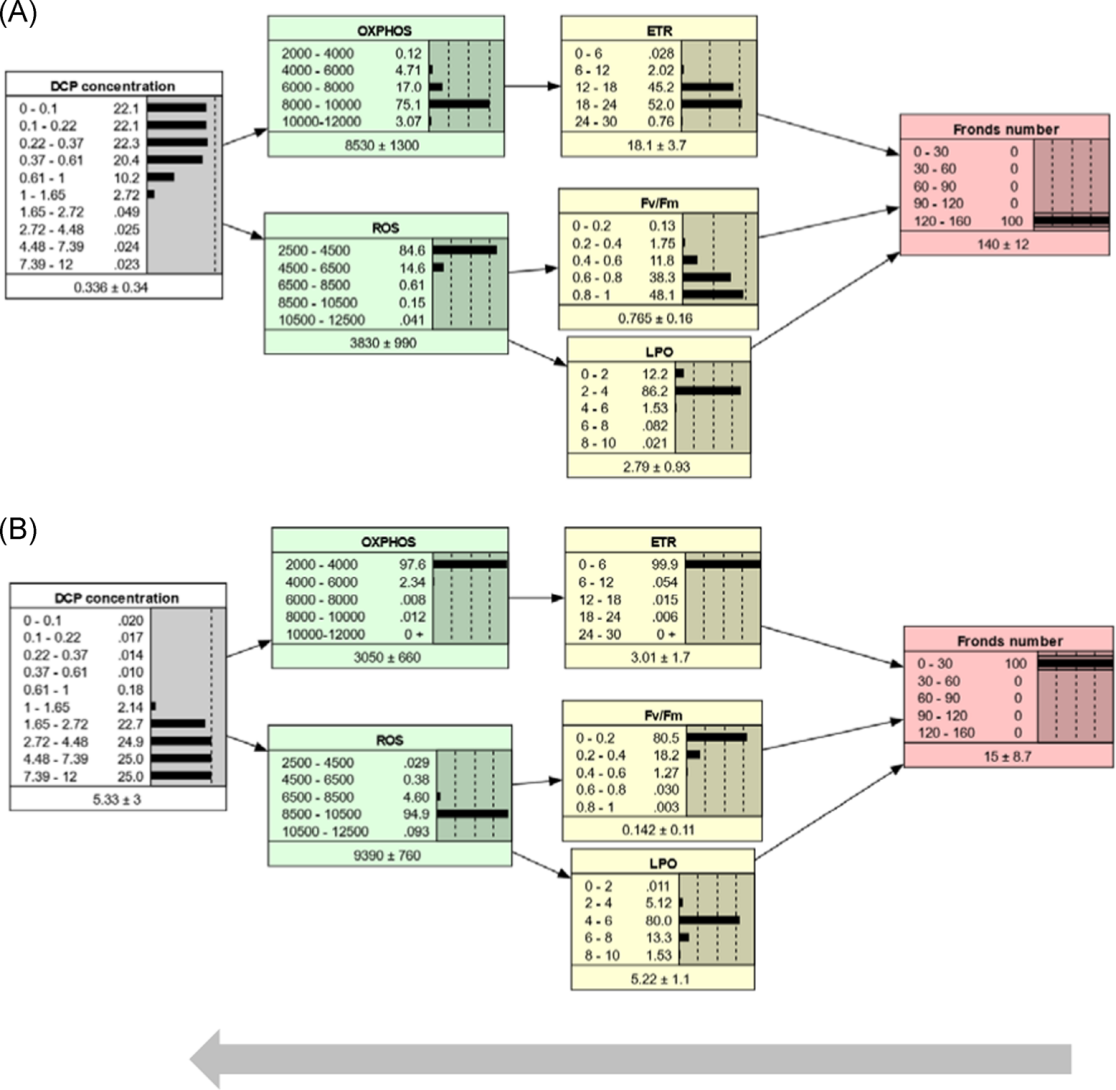
Diagnostic inference. The AOP‐BN model is run backwards from the adverse outcome node (fronds number): high fronds number (weakly adverse outcome) (**A**); low fronds number (strongly adverse outcome) (**B**). For more explanation, see Figure 4. AOP = adverse outcome pathway; BN = Bayesian network; DCP = 3,5‐dichlorophenol; ETR = electron transfer rate; Fv/Fm = maximum quantum yield of photosystem II; LPO = lipid peroxidation; OXPHOS = oxidative phosphorylation; ROS = reactive oxygen species.

**Figure 6:**
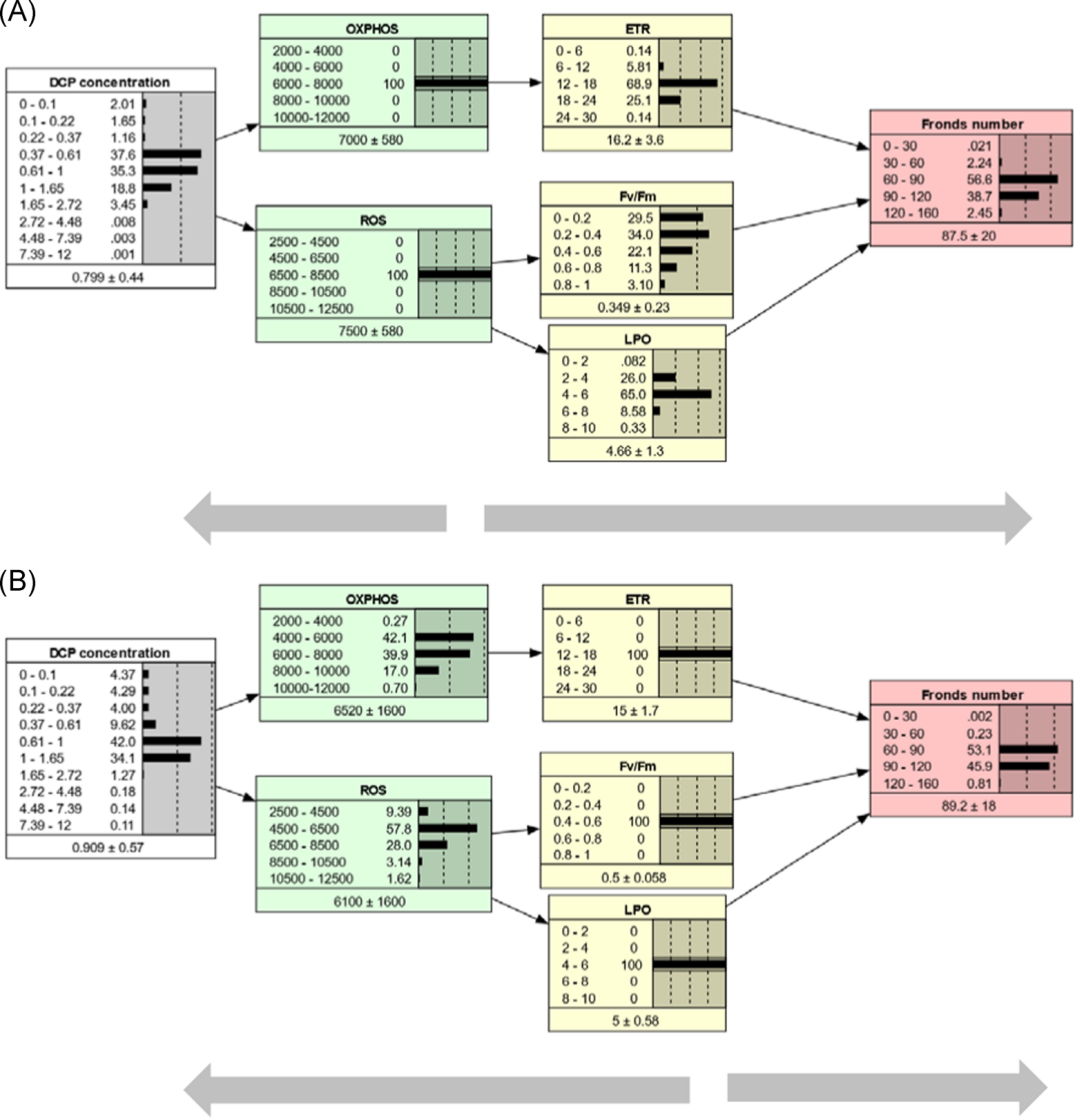
Combination of prognostic and diagnostic inference. The AOP‐BN model is run omnidirectionally from intermediate nodes: from molecular initiating events (**A**); from key events (**B**). For more explanation, see Figure 4. AOP = adverse outcome pathway; BN = Bayesian network; DCP = 3,5‐dichlorophenol; ETR = electron transfer rate; Fv/Fm = maximum quantum yield of photosystem II; LPO = lipid peroxidation; OXPHOS = oxidative phosphorylation; ROS = reactive oxygen species.

In the rest of this paper, the AOP‐BN version with combination Rule 2 is used to describe the behavior of the qAOP network, unless otherwise specified. A parameterized BN model can be run in different directions by setting evidence (i.e., set 100% probability of 1 state) for nodes in different locations, as exemplified by Figures 4, 5, and 6. The AOP‐BN can be run forward from the stressor node (prognostic, Figure 4), backward from the AO node (diagnostic, Figure 5), or omnidirectionally from any of the MIEs or KEs (Figure 6). The BN software provides a mean value for each node, calculated as the sum of each interval's midpoint weighted by its probability. Inspection of these mean node values can facilitate the comparison of predictions across different scenarios. Nevertheless, the full posterior probability distributions are more relevant than the means for a probabilistic risk assessment.

The prognostic model runs for 3 different stressor levels (Figure 4) show that the increasing DCP concentration resulted in decreased growth (fronds number) of *L. minor*, as expected. The predictions from the prognostic model runs can indicate the risk of exceeding a given AO threshold for different levels of the stressor. For example, assume a threshold of 90 fronds as a severe reduction of growth. At a low level of DCP (0.22–0.37 mg/L), the probability of a severely AO (<90 fronds) is less than 12% (Figure 2). At the intermediate DCP level (0.61–1 mg/L), the probability of a severely AO has increased to 31.3%, whereas at the high DCP level (1.65–2.72 mg/L), the probability is almost 97%. Increases in DCP concentration beyond this interval did not cause substantial further reduction of fronds number.

The predicted mean fronds number ranged from 54 at high stressor level to 109 at low stressor level, which is a narrower range than the observed number of fronds: average from 12 to 116 fronds for the range of DCP concentration. The ability of the AOP‐BN to predict the most profound AO (lowest fronds numbers) is limited by the lack of observed KE values for the 2 highest stressor concentrations (due to plant death). More generally, the AOP‐BN's performance when run from the stressor node is better for the intermediate stressor levels than for the extremely high or low stressor levels (Supplemental Data Figure S4).

The sensitivity of the AOP‐BN is described in Table 4. Pathway I (initiated by OXPHOS, Figure 1B) started affecting growth at lower DCP concentrations than Pathway II (initiated by ROS) (see Supplemental Data Figure S3). This result is consistent with the description of the qualitative AOP (Xie et al. 2018). Nevertheless, the ROS node had only marginally lower influence than OXPHOS on the AO (Table 4; 32.9% vs 33.3% mutual information under the selected combination Rule 2). This suggests that the ROS pathway also plays an important role in this AOP network, even though it reduces the overall accuracy of the AOP‐BN (Table 5; described later in this section). The contribution of the ROS pathway to the predicted AO might be more important at higher stressor levels, but such details are not revealed by this sensitivity analysis. The 4 alternative combination rules resulted in large variation in the sensitivity of the AO to the different parent nodes, with no systematic pattern (Table 4). This suggests that the choice of combination rule for integrating pathways in a quantitative AOP network can have a strong influence on the model's performance. The weighting of multiple pathways in an AOP‐BN should ideally be estimated directly from the data or validated by external data, whenever there are sufficient data.

The diagnostic run of the AOP‐BN backward from the AO node through the KEs and MIEs to the stressor node exemplifies a unique property of BN models (Kjærulff and Madsen 2008). The interpretation is less straightforward than for prognostic inference, but can be illustrated by 2 scenarios, representing the least AO (120–160 fronds, Figure 5A) and the most AO (0–30 fronds, Figure 5B). For a given target of the AO node, the posterior probabilities of the stressor states can be used to indicate the most likely range of stressor concentrations that must not be exceeded. In our example, given the target of 120 to 160 fronds (Figure 5A), the 4 stressor intervals below 0.61 mg/L all have approximately the same posterior probability (20.4%–22.1%), whereas the range 0.61 to 1 mg/L has only 10.2% probability, and the higher stressor intervals each have less than 0.5% probability. In terms of cumulative probability, if the regulatory protective or management target is the least AO, there is only 13% probability that the stressor can exceed 0.61 mg/L and only 3% probability that the stressor can exceed 1 mg/L. The equal posterior distribution of the 4 lowest stressor states reflects the low sensitivity of the model to stressor changes in this range (Supplemental Data Figure S4); a more sensitive model would more likely show a steeper increase in probability also for the lower stressor intervals.

When running the AOP‐BN backward from the AO of largest magnitude, conversely, the posterior probabilities of the stressor states show the range of stressor intervals that are most likely to cause this extreme state. In our example (Figure 5B), the 4 highest DCP intervals (1.65–12 mg/L) each have close to 25% probability. This implies that any concentration above 1.65 mg/L is equally likely to result in the most AO of 0 to 30 fronds. The AOP‐BN shows low sensitivity to changes in stressor level above 2.7 mg/L (Supplemental Data Figure S4). At this stressor level, OXPHOS has reached its lowest interval, whereas the next stressor interval (2.72–4.48 mg/L) results in a slight increase in ROS. As mentioned, the low model sensitivity at high stressor levels is related to the lack of observations of most variables (Supplemental Data Table S1) because of mortality. Although the target node has low sensitivity to changes in the highest stressor intervals, the AOP‐BN can still give an indication of safe versus unsafe stressor levels, accounting for the effects and related uncertainties throughout all 3 pathways. In our case study, this diagnostic inference has limited usefulness because growth inhibition may also be a result of many other MoAs that are not properly characterized and thus not included in the model. Nevertheless, the fact that BNs allow for backward running can be useful for more thoroughly developed AOP‐BNs based on data‐rich cases.

Finally, the AOP‐BN model can be run omnidirectionally from the MIEs (Figure 6A) or from the KEs (Figure 6B), or by a combination of these. In these examples, the middle interval was chosen for each node of the MIEs (Figure 6A) or KEs (Figure 6B), respectively. This example illustrates that a parameterized AOP‐BN can be used for quantitative prediction of the AO from measured events at various steps along the AOP continuum. The resulting posterior probability distributions of the AO were quite similar in the 2 cases, but running the AOP‐BN from the KERs compared to the MIEs resulted in a slightly lower standard deviation of the AO (Figure 6). In general, more precise AO predictions can be expected when the AOP‐BN is instantiated with observation closer to the AO node.

The performance of the AOP‐BN in terms of accuracy was more systematically evaluated by running the model from the stressor, MIE and KE nodes respectively, and comparing the predicted AO states with the observed fronds number (Table 5). The accuracy rate was calculated as the percent of cases in which the AO interval with the highest posterior probability was consistent with the observed interval, given the 4 alternative combination rules and the 3 alternative resolutions of the AO node (Table 3). Combination Rule 4, which represents Pathway I only (Figure 1B), generally resulted in higher accuracy than 3 other combination rules. Thus, the high uncertainty introduced by some of the estimated KERs in Pathways IIa and IIb (Figure 2) reduced the overall accuracy of the AO node. This also reflects a general property of BNs: Introducing more parent nodes will increase the variability of the child node, which accumulates the uncertainty of the parent nodes. The observation can also be interpreted in an toxicological context because the most sensitive pathway (Pathway I) is expected to be perturbed at the lowest concentrations, whereas increase in the exposure at intermediate to high concentrations would be expected to perturb an increasing number of pathways, of which one or more may not be properly characterized quantitatively and/or quantitatively and thus provide a poorer fit. This reduction in prediction power will be of largest relevance in high‐exposure scenarios, potentially reflecting either extreme exposures to single stressors or the combined effects of multiple chemicals interacting with the same MIE and sharing the same toxicological mechanism. However, an external validation with new data would likely result in lower accuracy than the current interval validation, even for the single‐pathway AOP (combination Rule 4). Because the present study focuses on the behavior of the whole network model, the performance of the BN with combination Rules 1 to 3 was inspected more closely.

Combination Rules 1 to 3 did not yield much difference in performance: Combination Rules 1 and 2 gave identical accuracy rates, whereas Rule 3 differed by 1 more wrong case (–5.6 percentage points) in 1 situation and 1 more correct case in 2 other situations.

Running the model from a node closer to the AO typically resulted in higher accuracy. For example, for combination Rule 2 with required precision of 3 states for the AO, the accuracy increased from 62.5% (model run from stressor) to 77.8% (run from MIEs) and further to 83.3% (run from KEs). In general, predictions based on measured variables (KEs) closer to the AO are likely to give more accurate (as well as precise) prediction than are predictions based on MIEs or earlier KEs. This observation provides confidence in the choice of endpoints used in populating both the qualitative and the quantitative AOP, given that coherence is expected to be largest between events close to each other and sensitivity of response should increase toward the MIE (Becker et al. 2015). In cases where measured values are only available for variables located early along the AOP continuum, the AOP‐BN can still be used for predicting a probability distribution of the AO, but possibly with lower accuracy and precision.

Lowering the resolution of the AO node also increased the accuracy rate. When the model performance was assessed by all 5 states of the AO, the accuracy rate was only in the range 37.5% to 61.5% for the first 3 combination rules. When the resolution was reduced to 3 states, the accuracy rate increased to the range 62.5% to 83.3%. Finally, with a binary AO node, the accuracy reached 94.4% to 100%. Thus, for applications of AOP‐BNs in which a low resolution (2–3 states) of the AO node is sufficient, a higher accuracy can be expected.

### Relevance of AOP‐BNs for environmental risk assessment

Characterizing the quantitative relationship between the MIE, KE, and AO and their KERs is a key feature in providing plausible causal relationships between the individual components of the AOP (Becker et al. 2015). Because AOPs are by definition chemically agnostic, they cannot be used directly to assess ecological risk, which should consider the effect of a stressor in relation to the exposure. The reliable development of quantitative relationships between the individual steps of the AOP, independent of the stressor perturbing the MIE, is thus a significant contribution toward more extensive use of mechanistic information for risk assessment purposes. Quantitative knowledge of the relationship between perturbed MIEs, KEs, and regulatory‐relevant AOs has potentially large relevance for how we assess the cumulative risk of multiple chemicals displaying similar MoA, which should be expected to share common AOPs. The AOP‐BNs offer an attractive alternative to other quantitative approaches based on point estimates (single values with confidence intervals), by explicitly modeling the uncertainty of each component and letting this uncertainty propagate throughout the entire AOP continuum or network.

With the probabilistic approach described in our study, we can refine the commonly used risk assessment approach, which is based on the calculation of a single‐value risk quotient (RQ) as the ratio exposure/effect. The binary approach—considering whether the risk quotient exceeds the value 1—has strong traditions in prospective risk assessment such as chemical safety assessment, and is typically the requirement for decision making, for example, for chemical product registration (Hunka et al. 2015). Our example shows that an AOP‐BN model can provide a probabilistic prediction of a binary target node with high accuracy. Such a probabilistic prediction can be more informative than the single‐number outcome of a traditional risk assessment (Carriger and Barron 2020). For example, the AOP‐BN combined with exposure data can quantify the probability of crossing a given AO threshold (e.g., below 90 fronds) to 12% at a low stressor level (Figure 4A) and to 43% at an intermediate stressor level (Figure 4B).

Although traditional regulatory risk assessment with a binary outcome is convenient for prospective chemical safety assessment, this binary approach has been criticized for being too conservative for environmental assessments, such as retrospective ecological risk assessment and chemical status assessment according to the Water Framework Directive (WFD; EC 2000) (Hunka et al. 2015; Posthuma et al. 2019). The problem with overconservative classification is exacerbated when strict combination rules are used for combined assessment of multiple stressors or multiple endpoints, and the tendency of too strict assessments further increases with higher levels of uncertainty (Moe et al. 2015). Probabilistic approaches, which can provide a probability distribution rather than a single value for the target variable, have therefore been advocated to better account for uncertainty in complex assessment systems. The BN implementation of the Relative Risk Model (BN‐RRM; Landis this issue) has been applied for this purpose in many different locations (Cains and Henshel this issue; Mitchell et al. this issue; Wade et al. this issue). Some of these applications include AOPs as integrated components of the BN model (Landis et al. 2020). Another recent example of multilevel endpoint assessment is based on species sensitivity distributions (SSDs) (Posthuma et al. 2019). Although ecological risk assessment based on SSDs typically calculated only a percentage of the curve (e.g., the lower 5% of species affected), Posthuma et al. (2019) converted the whole SSD curve into a 5‐level scale, representing different intervals of the SSD. This scale parallels the 5‐level ecological status class system of the WFD, which has been successfully incorporated in BN models for scenario assessments (e.g., Moe et al. 2016). The current AOP‐BN approach could easily be extended to incorporate an SSD as a multispecies, multistate endpoint node, given the relevant toxicity data.

The validation of the AOP‐BN model presented here showed that the accuracy of model predictions was relatively low for the 5‐state resolution of the AO node (61%–78% accuracy for predictions based on MIEs or KEs), but somewhat better for the 3‐state resolution (78%–89% accuracy). A higher accuracy rate may be desirable for an AOP‐BN model to be a reliable component of a risk assessment, but the performance will often be limited by the extent of the data set. The deviation between observed and predicted values in the lowest and highest ends of the stressor gradient (Supplemental Data Figure S4) is related to the high variability of simulated values for some KERs, for example, in ROS → Fv/Fm (Figure 2E). The variability of the measured values reflects partly aleatoric uncertainty (i.e., stochastic variation) and partly epistemic uncertainty (i.e., incomplete knowledge). It is commonly assumed that only the latter type of uncertainty can be reduced by additional measurements or improved modeling methods (Sahlin et al. this issue). Data resulting from toxicity testing typically contain a low number of stressor concentrations in replicates, but if the data are intended for regression modeling, the experimental units should preferably be distributed along the stressor gradient instead of being grouped into replicates (Fox and Landis 2016). In our case, the original data set (Supplemental Data Table S1) would have been more informative if the 24 experimental units were distributed as 24 points along the stressor concentration gradient, instead of being distributed at 6 concentrations with 3 (pseudo)replicates each. However, if more relevant data become available from other sources, these could be combined with the original data set in a hierarchical regression model (Gronewold and Borsuk 2010; Kotamäki et al. 2015) to update the CPTs, which could result in improved precision as well as accuracy. An independent data set could alternatively be used for a more thorough external validation of the AOP‐BN, which would allow assessment of performance to other chemicals interacting with the same MiEs and sharing the same set of AOPs (e.g., within the chemical applicability domain of the AOPs).

For the AOP‐BN model presented here, we have focused on the methodological aspects of quantification and validation, rather than on the usability of the results for risk assessment. All variables were therefore quantified with original values and units to aid the selection of the most appropriate statistical model. Nevertheless, all variables can easily be converted, for example, to percentage of deviation from a reference value or a management target, to make the model predictions more readily applicable for a risk assessment. Although our case study had a macrophyte as the model species for DCP with growth as the endpoint, the method is applicable to any taxa, endpoints, and stressor of concern.

Quantification of AOPs by the empirical approach described here will typically be more feasible than by mechanistic modeling methods based on systems biology (Schultz and Watanabe 2018), given that our approach requires fewer assumptions and can make use of the data more efficiently (Zgheib et al. 2019). In comparison with the other mentioned studies that have applied BN modeling for qAOPs, our study combines different benefits from the other studies. Firstly, we model more than 2 states of the AO (cf. Landis et al. 2020). Secondly, we model a network that combines several AOP chains (cf. Burgoon et al. 2019). Thirdly, we apply both Bayesian regression and Bayesian network modeling (cf. Zgheib et al. 2019) in an integrated approach.

## CONCLUSIONS AND OUTLOOK

Our study has demonstrated how an AOP network can be quantified based on a small data set, by taking advantage of both Bayesian regression analysis and Bayesian network modeling to combine several information sources: experimental data, established dose–response functions, and expert knowledge. The quantification of each KER by CPTs is consistent with the modular approach recommended by Foran et al. (2019). The quantified AOP‐BN can be applied to predict the probability and severity of AOs from measured responses of MIEs or KEs, in cases where it is impossible or impractical to measure the AOs directly.

Although we chose a Bayesian regression method for flexible modeling of the dose– and response–response relationships, similar results could be obtained by the more common frequentist dose–response regression models (Ritz et al. 2015). Thus, this relatively simple approach of regression modeling combined with BN modeling can be recommended as a first step for quantification of AOPs and AOP networks based on limited data sets. The use of regression models for quantifying the CPTs, as opposed to for count of observations or expert judgment only, allows for a higher resolution of the nodes (although our example was limited to only 5 states for simplicity). Hence, the loss of information due to discretization of variables, which is an inherent shortcoming of BN modeling, can be mitigated by combination with regression modeling.

The modeling approach presented here can still be improved in several ways, if sufficient data sets become available. For example, the issue of pseudoreplication, which must be handled when defining response–response relationships, could be modeled more explicitly. The selection and parametrization of the individual dose–response functions could be refined by more extensive statistical model selection procedures. Moreover, an optimal weighting of multiple pathways could be estimated.

The AO endpoint of AOP‐BN model may be further extended from the individual response level to include population‐level responses. Although BN models are not commonly used for dynamic modeling such as population dynamics, the outcome of a population model can be incorporated as an AO (Mitchell et al. this issue). For species like *L. minor*, for which mechanistic effect and population models are already developed and are being applied for regulatory purposes (Schmitt et al. 2013; Hommen et al. 2016), incorporating an additional AO node based on population modeling can be a straightforward next step. Furthermore, Murphy et al. (2018) and Schultz and Watanabe (2018) have proposed the use of dynamic energy budget (DEB) for linking quantitative AOPs with population‐level responses, using the individual organisms as a “pivot” connecting suborganismal processes to higher level ecological processes. In the opposite end, the AOP‐BN can be linked to a stressor source through an aggregate exposure pathway, which is a conceptual framework to characterize relationships between stressor source, exposure route, internal exposure (e.g., toxicokinetics) and resulting target exposure (Hines et al. 2018; Tan et al. 2018). Given the modular and flexible structure of BNs, an AOP‐BN can also be extended to handle multiple stressors, where different types of interactions can be implemented in the CPTs, or to predict AOs of multiple biological endpoints.

## Disclaimer

The authors declare no conflict of interest. The peer review for this article was managed by the Editorial Board without the involvement of SJ Moe or WG Landis.

## SUPPLEMENTAL DATA

**Figure S1.** Proposed AOP #245.

**Figure S2.** Examples of estimated dose–response (a, d) and response–response (b, c, e–h) curves.

**Figure S3.** Integration of the 3 adverse outcome pathways with 4 alternative combination rules.

**Figure S4.** Internal validation of the AOP‐BN model.

**Table S1.** Experimental data set used for quantification of the AOP‐BN

**Table S2.** Conditional probability tables for all dose–response and response–response relationships

## Supporting information

This article contains online‐only Supplemental Data.

Supporting information.

## Data Availability

All background data are provided in Supplemental Data Table S1. For the regression models (section *Bayesian regression analysis and simulation*), all model code is available upon request from corresponding author S Jannicke Moe (jmo@niva.no). The full model specification of the Bayesian network (section *Quantification of the AOP‐BN*), that is, the conditional probability tables, is provided in Supplemental Data Tables S2a to S2i.
