# Supplementary material for: Quantification of an Adverse Outcome Pathway Network by Bayesian Regression and Bayesian Network Modeling
Source: Integr Environ Assess Manag. 2020 Oct 23;17(1):147–64. doi: 10.1002/ieam.4348 (PMC7820971; doi:10.1002/ieam.4348)
Supplement: Supplementary file 1 — Supporting information. [file IEAM-17-147-s001.docx]

**Supplementary material to IEAM-2020-100-SS:**"**Quantification of an Adverse Outcome Pathway network by Bayesian regression and Bayesian network modelling"**

**Table S1.** Experimental dataset used for quantification of the AOP-BN. Within each stressor treatment level (DCP), the three repeated measurements of each response variable have been randomly ordered in this table. The missing values for some variables at concentrations >= 4 mg/L are due to mortality of the plants. More details are provided by Xie et al. (2018).

| **DCP (mg/L)** | **OXPHOS**  **(emitted photons / 100 ms)** | **ETR (µmol/ (m^2^ s))** | **ROS (emitted photons / 100 ms)** | **Fv/Fm (ratio of counts)** | **LPO (µmol/g)** | **Fronds number** |
| --- | --- | --- | --- | --- | --- | --- |
| 0 | 9476 | 20.346 | 3635 | 0.67 | 2.25 | 105 |
| 0 | 9568 | 20.536 | 4380 | 0.663 | 3.36 | 122 |
| 0 | 10580 | 19.416 | 2889 | 0.665 | 2.33 | 122 |
| 0.5 | 7671 | 17.099 | 3285 | 0.685 | 2.79 | 114 |
| 0.5 | 7479 | 16.908 | 4443 | 0.676 | 2.66 | 112 |
| 0.5 | 8633 | 14.199 | 5601 | 0.677 | 2.27 | 122 |
| 1 | 5774 | 11.814 | 6124 | 0.663 | 4.35 | 100 |
| 1 | 5583 | 11.131 | 5542 | 0.662 | 3.96 | 102 |
| 1 | 5379 | 11.6 | 5833 | 0.646 | 3.29 | 94 |
| 1.5 | 4122 | 8.522 | 7110 | 0.54 | 4.92 | 82 |
| 1.5 | 4243 | 8.264 | 6633 | 0.473 | 4.68 | 79 |
| 1.5 | 3988 | 7.592 | 6157 | 0.484 | 5.14 | 77 |
| 2 | 3620 | 2.094 | 12159 | 0.224 | 4.45 | 50 |
| 2 | 3372 | 1.904 | 7341 | 0.247 | 4.9 | 47 |
| 2 | 4077 | 3.371 | 9750 | 0.231 | 5.11 | 45 |
| 3 | 3018 | 0.001 | 8636 | 0.028 | 4.94 | 17 |
| 3 | 3473 | 0.504 | 9240 | 0.022 | 3.85 | 18 |
| 3 | 3269 | 0.437 | 8032 | 0.048 | 4.43 | 18 |
| 4 | NA | 0 | NA | 0 | NA | 15 |
| 4 | NA | 0 | NA | 0.02 | NA | 12 |
| 4 | NA | 0 | NA | 0 | NA | 13 |
| 8 | NA | NA | NA | NA | NA | 12 |
| 8 | NA | NA | NA | NA | NA | 12 |
| 8 | NA | NA | NA | NA | NA | 12 |


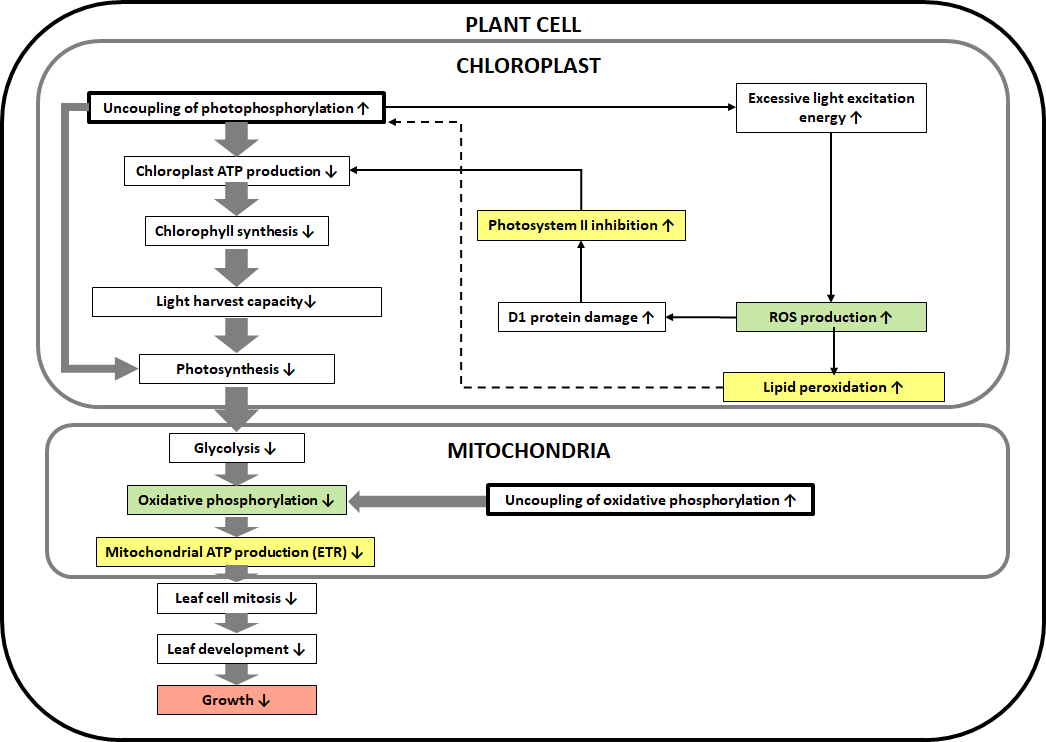


**Figure S1.** Proposed AOP #245: a network of Adverse Outcome Pathway (AOP) linking the mode of action of the stressor 3,5-dichlorophenol (DCP) to adverse outcomes in *Lemna minor*. After Xie et a. (2018).

**
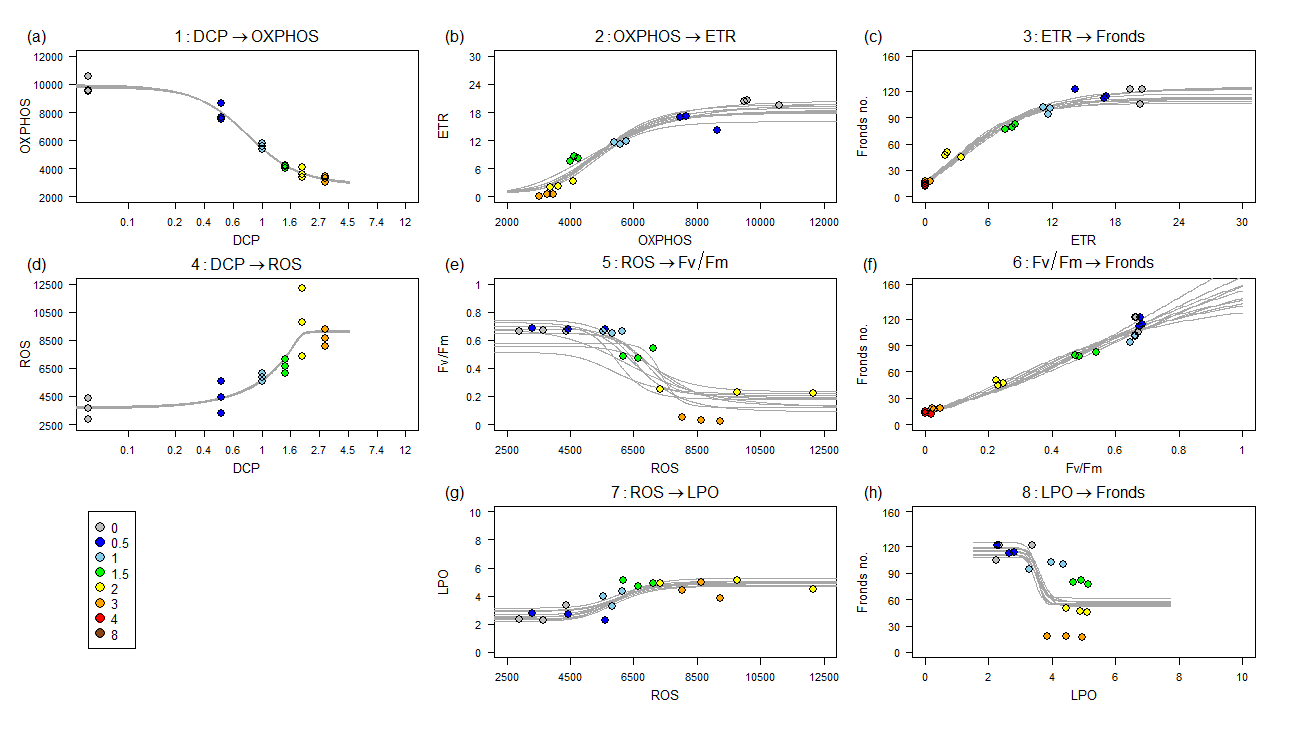
**

**Figure S2**. Examples of quantified causal relationships: dose-response curves (a,d) and response-response curves (all others) estimated from the experimental data (Table S1) by Bayesian regression models. In each plot, the curves are 10 realisations of the estimated relationship, based on 10 randomly draws from the joint posterior distribution of the parameters. For more details, see Figure 2.

**Conditional probability tables for dose-response and response-response relationships**

**Table S2.** Conditional probability tables (CPTs) for dose-response and response-response relationships. The individual CPTs are labelled by letters (a), (b) etc., corresponding to the individual plots of Figure 2. The probabilities are given as proportions. Note that the table format below (row sums = 1) differs from the table format in Figure 3 (column sums = 100%).

**(a)** CPT for relationship 1: DCP → OXPHOS. Note the discretisation of DCP is on logarithmic scale.

| **OXPHOS DCP** | **[2000, 4000)** | **[4000, 6000)** | **[6000, 8000)** | **[8000, 10000)** | **[10000, 12000]** | **Count** |
| --- | --- | --- | --- | --- | --- | --- |
| **[0, 0.1)** | 0 | 0 | 0 | 0.9 | 0.1 | **90** |
| **[0.1, 0.223)** | 0 | 0 | 0 | 1 | 0 | **100** |
| **[0.223, 0.368)** | 0 | 0 | 0 | 1 | 0 | **120** |
| **[0.368, 0.607)** | 0 | 0 | 0.43 | 0.57 | 0 | **200** |
| **[0.607, 1)** | 0 | 0.342 | 0.656 | 0 | 0 | **330** |
| **[1, 1.65)** | 0.013 | 0.987 | 0 | 0 | 0 | **540** |
| **[1.65, 2.72)** | 0.966 | 0.034 | 0 | 0 | 0 | **890** |
| **[2.72, 4.48)** | 1 | 0 | 0 | 0 | 0 | **1470** |
| **[4.48, 7.39)** | 1 | 0 | 0 | 0 | 0 | **2420** |
| **[7.39, 12]** | 1 | 0 | 0 | 0 | 0 | **3840** |
|  |  |  |  |  |  |  |

**(b)** CPT for relationship 2: OXPHOS → ETR.

| **ETR OXPHOS** | **[0, 6)** | **[6, 12)** | **[12, 18)** | **[18, 24)** | **[24, 30]** | **Count** |
| --- | --- | --- | --- | --- | --- | --- |
| **[2000, 4000)** | 0.905 | 0.095 | 0 | 0 | 0 | **1440** |
| **[4000, 6000)** | 0.178 | 0.536 | 0.278 | 0.0076 | 0 | **1440** |
| **[6000, 8000)** | 0.00069 | 0.058 | 0.690 | 0.251 | 0.00069 | **1440** |
| **[8000, 10000)** | 0 | 0.008 | 0.496 | 0.491 | 0.0056 | **1440** |
| **[10000, 12000]** | 0 | 0.006 | 0.367 | 0.608 | 0.018 | **1440** |

**(c)** CPT for relationship 3: ETR → Fronds number.

| **Fronds ETR** | **[0, 30)** | **[30, 60)** | **[60, 90)** | **[90, 120)** | **[120, 160]** | **Count** |
| --- | --- | --- | --- | --- | --- | --- |
| **[0, 6)** | 0.264 | 0.524 | 0.209 | 0.0041 | 0 | **1950** |
| **[6, 12)** | 0 | 0.010 | 0.488 | 0.490 | 0.012 | **1950** |
| **[12, 18)** | 0 | 0 | 0.038 | 0.785 | 0.177 | **1940** |
| **[18, 24)** | 0 | 0 | 0.012 | 0.650 | 0.337 | **1950** |
| **[24, 30]** | 0 | 0 | 0.010 | 0.585 | 0.405 | **1950** |

**(d)** CPT for relationship 4: DCP → ROS.

| **ROS DCP** | **[2500, 4500)** | **[4500, 6500)** | **[6500, 8500)** | **[8500, 10500)** | **[10500, 12500]** | **Count** |
| --- | --- | --- | --- | --- | --- | --- |
| **[0, 0.1)** | 1 | 0 | 0 | 0 | 0 | **90** |
| **[0.1, 0.223)** | 1 | 0 | 0 | 0 | 0 | **100** |
| **[0.223, 0.368)** | 1 | 0 | 0 | 0 | 0 | **120** |
| **[0.368, 0.607)** | 0.860 | 0.140 | 0 | 0 | 0 | **200** |
| **[0.607, 1)** | 0.00606 | 0.994 | 0 | 0 | 0 | **330** |
| **[1, 1.65)** | 0 | 0.433 | 0.567 | 0 | 0 | **540** |
| **[1.65, 2.72)** | 0 | 0 | 0.170 | 0.830 | 0 | **890** |
| **[2.72, 4.48)** | 0 | 0 | 0 | 1 | 0 | **1470** |
| **[4.48, 7.39)** | 0 | 0 | 0 | 1 | 0 | **2420** |
| **[7.39, 12]** | 0 | 0 | 0 | 1 | 0 | **3840** |

**(e)** CPT for relationship 5: ROS → LPO.

| **LPO ROS** | **[0, 2)** | **[2, 4)** | **[4, 6)** | **[6, 8)** | **[8, 10]** | **Count** |
| --- | --- | --- | --- | --- | --- | --- |
| **[2500, 4500)** | 0.106 | 0.875 | 0.019 | 0 | 0 | **1220** |
| **[4500, 6500)** | 0.019 | 0.724 | 0.247 | 0.011 | 0 | **1230** |
| **[6500, 8500)** | 0 | 0.260 | 0.652 | 0.085 | 0.0025 | **1219** |
| **[8500, 10500)** | 0.00081 | 0.199 | 0.679 | 0.109 | 0.012 | **1229** |
| **[10500, 12500]** | 0 | 0.189 | 0.669 | 0.133 | 0.010 | **1219** |

**(f)** CPT for relationship 6: LPO → Fronds number. Note: the highest interval of LPO had zero simulated values; the probabilities were thererefore extrapolated from the penultimate interval.

| **Fronds LPO** | **[0, 30)** | **[30, 60)** | **[60, 90)** | **[90, 120)** | **[120, 160]** | **Count** |
| --- | --- | --- | --- | --- | --- | --- |
| **[0, 2)** | 0 | 0 | 0.0086 | 0.609 | 0.383 | **810** |
| **[2, 4)** | 0 | 0.044 | 0.231 | 0.516 | 0.209 | **3220** |
| **[4, 6)** | 0.00031 | 0.612 | 0.388 | 0 | 0 | **3210** |
| **[6, 8)** | 0 | 0.659 | 0.341 | 0 | 0 | **2760** |
| **[8, 10]** | 0 | 0.659 | 0.341 | 0 | 0 | **0** |

**(g)** CPT for relationship 7: ROS → Fv/FM.

| **Fv/Fm ROS** | **[0, 0.2)** | **[0.2, 0.4)** | **[0.4, 0.6)** | **[0.6, 0.8)** | **[0.8, 1]** | **Count** |
| --- | --- | --- | --- | --- | --- | --- |
| **[2500, 4500)** | 0.024 | 0.093 | 0.228 | 0.343 | 0.312 | **1220** |
| **[4500, 6500)** | 0.047 | 0.172 | 0.281 | 0.315 | 0.185 | **1230** |
| **[6500, 8500)** | 0.295 | 0.340 | 0.221 | 0.113 | 0.030 | **1220** |
| **[8500, 10500)** | 0.520 | 0.296 | 0.126 | 0.046 | 0.013 | **1230** |
| **[10500, 12500]** | 0.561 | 0.272 | 0.117 | 0.041 | 0.0082 | **1220** |

**(h)** CPT for relationship 8: Fv/FM → Fronds number.

| **Fronds Fv/Fm** | **[0, 30)** | **[30, 60)** | **[60, 90)** | **[90, 120)** | **[120, 160]** | **Count** |
| --- | --- | --- | --- | --- | --- | --- |
| **[0, 0.2)** | 0.703 | 0.298 | 0 | 0 | 0 | **2000** |
| **[0.2, 0.4)** | 0.018 | 0.710 | 0.270 | 0.0030 | 0 | **2000** |
| **[0.4, 0.6)** | 0 | 0.026 | 0.626 | 0.346 | 0.0025 | **2000** |
| **[0.6, 0.8)** | 0 | 0 | 0.034 | 0.618 | 0.348 | **2000** |
| **[0.8, 1]** | 0 | 0 | 0.00050 | 0.127 | 0.873 | **2000** |

**(i)** CPT for integrating the three pathways into the adverse outcome node Fronds by combination rule 2, after absorbtion of the intermediate Fronds nodes (Figure S5.1).

| **Parent nodes** | | | **Fronds number** | | | | |
| --- | --- | --- | --- | --- | --- | --- | --- |
| **ETR** | **Fv/Fm** | **LPO** | **[0, 30)** | **[30, 60)** | **[60, 90)** | **[90, 120)** | **[120, 160)** |
| **[0, 6)** | **[0, 0.2)** | **[0, 2)** | 1.11E-02 | 5.79E-01 | 4.03E-01 | 6.43E-03 | 2.87E-05 |
| **[0, 6)** | **[0, 0.2)** | **[2, 4)** | 4.11E-02 | 6.44E-01 | 3.11E-01 | 3.97E-03 | 1.65E-05 |
| **[0, 6)** | **[0, 0.2)** | **[4, 6)** | 1.80E-01 | 7.34E-01 | 8.60E-02 | 3.48E-04 | 1.72E-07 |
| **[0, 6)** | **[0, 0.2)** | **[6, 8)** | 1.86E-01 | 7.32E-01 | 8.17E-02 | 3.31E-04 | 1.68E-07 |
| **[0, 6)** | **[0, 0.2)** | **[8, 10]** | 1.86E-01 | 7.32E-01 | 8.17E-02 | 3.31E-04 | 1.68E-07 |
| **[0, 6)** | **[0.2, 0.4)** | **[0, 2)** | 8.45E-04 | 3.78E-01 | 5.91E-01 | 2.93E-02 | 1.12E-04 |
| **[0, 6)** | **[0.2, 0.4)** | **[2, 4)** | 8.90E-03 | 4.79E-01 | 4.94E-01 | 1.80E-02 | 7.04E-05 |
| **[0, 6)** | **[0.2, 0.4)** | **[4, 6)** | 6.95E-02 | 7.21E-01 | 2.08E-01 | 9.05E-04 | 1.37E-06 |
| **[0, 6)** | **[0.2, 0.4)** | **[6, 8)** | 7.35E-02 | 7.24E-01 | 2.01E-01 | 8.59E-04 | 1.24E-06 |
| **[0, 6)** | **[0.2, 0.4)** | **[8, 10]** | 7.35E-02 | 7.24E-01 | 2.01E-01 | 8.59E-04 | 1.24E-06 |
| **[0, 6)** | **[0.4, 0.6)** | **[0, 2)** | 1.58E-04 | 1.80E-01 | 7.21E-01 | 9.84E-02 | 3.73E-04 |
| **[0, 6)** | **[0.4, 0.6)** | **[2, 4)** | 9.35E-04 | 2.71E-01 | 6.62E-01 | 6.60E-02 | 2.53E-04 |
| **[0, 6)** | **[0.4, 0.6)** | **[4, 6)** | 1.07E-02 | 5.79E-01 | 4.05E-01 | 5.07E-03 | 2.35E-05 |
| **[0, 6)** | **[0.4, 0.6)** | **[6, 8)** | 1.14E-02 | 5.89E-01 | 3.95E-01 | 4.67E-03 | 2.13E-05 |
| **[0, 6)** | **[0.4, 0.6)** | **[8, 10]** | 1.14E-02 | 5.89E-01 | 3.95E-01 | 4.67E-03 | 2.13E-05 |
| **[0, 6)** | **[0.6, 0.8)** | **[0, 2)** | 3.43E-05 | 6.44E-02 | 6.98E-01 | 2.36E-01 | 1.48E-03 |
| **[0, 6)** | **[0.6, 0.8)** | **[2, 4)** | 7.17E-05 | 1.24E-01 | 7.02E-01 | 1.73E-01 | 9.72E-04 |
| **[0, 6)** | **[0.6, 0.8)** | **[4, 6)** | 6.20E-04 | 3.62E-01 | 6.07E-01 | 3.08E-02 | 1.20E-04 |
| **[0, 6)** | **[0.6, 0.8)** | **[6, 8)** | 6.55E-04 | 3.72E-01 | 5.99E-01 | 2.88E-02 | 1.13E-04 |
| **[0, 6)** | **[0.6, 0.8)** | **[8, 10]** | 6.55E-04 | 3.72E-01 | 5.99E-01 | 2.88E-02 | 1.13E-04 |
| **[0, 6)** | **[0.8, 1]** | **[0, 2)** | 1.23E-05 | 2.03E-02 | 6.26E-01 | 3.51E-01 | 2.76E-03 |
| **[0, 6)** | **[0.8, 1]** | **[2, 4)** | 3.44E-05 | 5.92E-02 | 6.75E-01 | 2.64E-01 | 1.79E-03 |
| **[0, 6)** | **[0.8, 1]** | **[4, 6)** | 1.68E-04 | 2.33E-01 | 7.05E-01 | 6.12E-02 | 2.16E-04 |
| **[0, 6)** | **[0.8, 1]** | **[6, 8)** | 1.74E-04 | 2.41E-01 | 7.01E-01 | 5.77E-02 | 2.07E-04 |
| **[0, 6)** | **[0.8, 1]** | **[8, 10]** | 1.74E-04 | 2.41E-01 | 7.01E-01 | 5.77E-02 | 2.07E-04 |
| **[6, 12)** | **[0, 0.2)** | **[0, 2)** | 2.60E-05 | 2.95E-02 | 7.60E-01 | 2.10E-01 | 7.53E-04 |
| **[6, 12)** | **[0, 0.2)** | **[2, 4)** | 1.01E-04 | 8.87E-02 | 7.69E-01 | 1.42E-01 | 4.37E-04 |
| **[6, 12)** | **[0, 0.2)** | **[4, 6)** | 6.31E-04 | 3.42E-01 | 6.45E-01 | 1.22E-02 | 4.32E-06 |
| **[6, 12)** | **[0, 0.2)** | **[6, 8)** | 6.64E-04 | 3.52E-01 | 6.36E-01 | 1.13E-02 | 4.21E-06 |
| **[6, 12)** | **[0, 0.2)** | **[8, 10]** | 6.64E-04 | 3.52E-01 | 6.36E-01 | 1.13E-02 | 4.21E-06 |
| **[6, 12)** | **[0.2, 0.4)** | **[0, 2)** | 2.48E-06 | 4.86E-03 | 5.68E-01 | 4.24E-01 | 2.90E-03 |
| **[6, 12)** | **[0.2, 0.4)** | **[2, 4)** | 1.80E-05 | 2.08E-02 | 6.60E-01 | 3.17E-01 | 1.81E-03 |
| **[6, 12)** | **[0.2, 0.4)** | **[4, 6)** | 1.43E-04 | 1.34E-01 | 8.11E-01 | 5.50E-02 | 3.32E-05 |
| **[6, 12)** | **[0.2, 0.4)** | **[6, 8)** | 1.51E-04 | 1.41E-01 | 8.08E-01 | 5.08E-02 | 3.01E-05 |
| **[6, 12)** | **[0.2, 0.4)** | **[8, 10]** | 1.51E-04 | 1.41E-01 | 8.08E-01 | 5.08E-02 | 3.01E-05 |
| **[6, 12)** | **[0.4, 0.6)** | **[0, 2)** | 3.37E-07 | 1.05E-03 | 3.21E-01 | 6.61E-01 | 1.71E-02 |
| **[6, 12)** | **[0.4, 0.6)** | **[2, 4)** | 1.84E-06 | 3.52E-03 | 4.38E-01 | 5.48E-01 | 1.06E-02 |
| **[6, 12)** | **[0.4, 0.6)** | **[4, 6)** | 2.10E-05 | 2.49E-02 | 7.72E-01 | 2.03E-01 | 5.72E-04 |
| **[6, 12)** | **[0.4, 0.6)** | **[6, 8)** | 2.24E-05 | 2.64E-02 | 7.80E-01 | 1.93E-01 | 5.19E-04 |
| **[6, 12)** | **[0.4, 0.6)** | **[8, 10]** | 2.24E-05 | 2.64E-02 | 7.80E-01 | 1.93E-01 | 5.19E-04 |
| **[6, 12)** | **[0.6, 0.8)** | **[0, 2)** | 7.01E-08 | 2.23E-04 | 1.34E-01 | 7.80E-01 | 8.55E-02 |
| **[6, 12)** | **[0.6, 0.8)** | **[2, 4)** | 1.54E-07 | 6.94E-04 | 2.29E-01 | 7.16E-01 | 5.45E-02 |
| **[6, 12)** | **[0.6, 0.8)** | **[4, 6)** | 1.40E-06 | 4.09E-03 | 5.54E-01 | 4.39E-01 | 3.02E-03 |
| **[6, 12)** | **[0.6, 0.8)** | **[6, 8)** | 1.49E-06 | 4.29E-03 | 5.65E-01 | 4.28E-01 | 2.85E-03 |
| **[6, 12)** | **[0.6, 0.8)** | **[8, 10]** | 1.49E-06 | 4.29E-03 | 5.65E-01 | 4.28E-01 | 2.85E-03 |
| **[6, 12)** | **[0.8, 1]** | **[0, 2)** | 2.74E-08 | 7.53E-05 | 5.10E-02 | 7.89E-01 | 1.60E-01 |
| **[6, 12)** | **[0.8, 1]** | **[2, 4)** | 8.20E-08 | 2.63E-04 | 1.22E-01 | 7.74E-01 | 1.04E-01 |
| **[6, 12)** | **[0.8, 1]** | **[4, 6)** | 5.28E-07 | 1.49E-03 | 4.04E-01 | 5.89E-01 | 5.51E-03 |
| **[6, 12)** | **[0.8, 1]** | **[6, 8)** | 5.54E-07 | 1.56E-03 | 4.15E-01 | 5.78E-01 | 5.28E-03 |
| **[6, 12)** | **[0.8, 1]** | **[8, 10]** | 5.54E-07 | 1.56E-03 | 4.15E-01 | 5.78E-01 | 5.28E-03 |
| **[12, 18)** | **[0, 0.2)** | **[0, 2)** | 2.14E-05 | 3.03E-03 | 5.23E-01 | 4.65E-01 | 9.62E-03 |
| **[12, 18)** | **[0, 0.2)** | **[2, 4)** | 7.91E-05 | 9.64E-03 | 6.32E-01 | 3.53E-01 | 5.53E-03 |
| **[12, 18)** | **[0, 0.2)** | **[4, 6)** | 3.35E-04 | 5.97E-02 | 8.43E-01 | 9.70E-02 | 5.91E-05 |
| **[12, 18)** | **[0, 0.2)** | **[6, 8)** | 3.46E-04 | 6.29E-02 | 8.44E-01 | 9.29E-02 | 5.77E-05 |
| **[12, 18)** | **[0, 0.2)** | **[8, 10]** | 3.46E-04 | 6.29E-02 | 8.44E-01 | 9.29E-02 | 5.77E-05 |
| **[12, 18)** | **[0.2, 0.4)** | **[0, 2)** | 1.60E-06 | 7.92E-04 | 2.48E-01 | 7.13E-01 | 3.83E-02 |
| **[12, 18)** | **[0.2, 0.4)** | **[2, 4)** | 1.73E-05 | 2.02E-03 | 3.87E-01 | 5.87E-01 | 2.40E-02 |
| **[12, 18)** | **[0.2, 0.4)** | **[4, 6)** | 1.35E-04 | 1.15E-02 | 7.76E-01 | 2.12E-01 | 4.74E-04 |
| **[12, 18)** | **[0.2, 0.4)** | **[6, 8)** | 1.43E-04 | 1.21E-02 | 7.84E-01 | 2.03E-01 | 4.30E-04 |
| **[12, 18)** | **[0.2, 0.4)** | **[8, 10]** | 1.43E-04 | 1.21E-02 | 7.84E-01 | 2.03E-01 | 4.30E-04 |
| **[12, 18)** | **[0.4, 0.6)** | **[0, 2)** | 3.07E-07 | 3.54E-04 | 5.87E-02 | 8.30E-01 | 1.11E-01 |
| **[12, 18)** | **[0.4, 0.6)** | **[2, 4)** | 1.82E-06 | 5.79E-04 | 1.51E-01 | 7.72E-01 | 7.73E-02 |
| **[12, 18)** | **[0.4, 0.6)** | **[4, 6)** | 2.08E-05 | 2.26E-03 | 5.31E-01 | 4.59E-01 | 8.15E-03 |
| **[12, 18)** | **[0.4, 0.6)** | **[6, 8)** | 2.23E-05 | 2.37E-03 | 5.46E-01 | 4.45E-01 | 7.35E-03 |
| **[12, 18)** | **[0.4, 0.6)** | **[8, 10]** | 2.23E-05 | 2.37E-03 | 5.46E-01 | 4.45E-01 | 7.35E-03 |
| **[12, 18)** | **[0.6, 0.8)** | **[0, 2)** | 6.67E-08 | 1.28E-04 | 1.39E-02 | 7.23E-01 | 2.63E-01 |
| **[12, 18)** | **[0.6, 0.8)** | **[2, 4)** | 1.39E-07 | 2.36E-04 | 4.36E-02 | 7.63E-01 | 1.93E-01 |
| **[12, 18)** | **[0.6, 0.8)** | **[4, 6)** | 1.20E-06 | 6.85E-04 | 2.30E-01 | 7.28E-01 | 4.12E-02 |
| **[12, 18)** | **[0.6, 0.8)** | **[6, 8)** | 1.27E-06 | 7.03E-04 | 2.41E-01 | 7.19E-01 | 3.88E-02 |
| **[12, 18)** | **[0.6, 0.8)** | **[8, 10]** | 1.27E-06 | 7.03E-04 | 2.41E-01 | 7.19E-01 | 3.88E-02 |
| **[12, 18)** | **[0.8, 1]** | **[0, 2)** | 2.38E-08 | 4.10E-05 | 5.34E-03 | 5.93E-01 | 4.02E-01 |
| **[12, 18)** | **[0.8, 1]** | **[2, 4)** | 6.62E-08 | 1.18E-04 | 1.63E-02 | 6.87E-01 | 2.97E-01 |
| **[12, 18)** | **[0.8, 1]** | **[4, 6)** | 3.17E-07 | 4.63E-04 | 9.07E-02 | 8.35E-01 | 7.40E-02 |
| **[12, 18)** | **[0.8, 1]** | **[6, 8)** | 3.28E-07 | 4.77E-04 | 9.52E-02 | 8.33E-01 | 7.10E-02 |
| **[12, 18)** | **[0.8, 1]** | **[8, 10]** | 3.28E-07 | 4.77E-04 | 9.52E-02 | 8.33E-01 | 7.10E-02 |
| **[18, 24)** | **[0, 0.2)** | **[0, 2)** | 2.13E-05 | 1.76E-03 | 4.22E-01 | 5.58E-01 | 1.81E-02 |
| **[18, 24)** | **[0, 0.2)** | **[2, 4)** | 7.87E-05 | 5.02E-03 | 5.31E-01 | 4.54E-01 | 1.04E-02 |
| **[18, 24)** | **[0, 0.2)** | **[4, 6)** | 3.33E-04 | 3.74E-02 | 7.88E-01 | 1.74E-01 | 1.12E-04 |
| **[18, 24)** | **[0, 0.2)** | **[6, 8)** | 3.44E-04 | 3.97E-02 | 7.93E-01 | 1.67E-01 | 1.09E-04 |
| **[18, 24)** | **[0, 0.2)** | **[8, 10]** | 3.44E-04 | 3.97E-02 | 7.93E-01 | 1.67E-01 | 1.09E-04 |
| **[18, 24)** | **[0.2, 0.4)** | **[0, 2)** | 1.60E-06 | 6.97E-04 | 1.90E-01 | 7.38E-01 | 7.22E-02 |
| **[18, 24)** | **[0.2, 0.4)** | **[2, 4)** | 1.72E-05 | 1.17E-03 | 3.09E-01 | 6.44E-01 | 4.54E-02 |
| **[18, 24)** | **[0.2, 0.4)** | **[4, 6)** | 1.35E-04 | 4.84E-03 | 6.69E-01 | 3.25E-01 | 9.01E-04 |
| **[18, 24)** | **[0.2, 0.4)** | **[6, 8)** | 1.42E-04 | 5.06E-03 | 6.78E-01 | 3.16E-01 | 8.16E-04 |
| **[18, 24)** | **[0.2, 0.4)** | **[8, 10]** | 1.42E-04 | 5.06E-03 | 6.78E-01 | 3.16E-01 | 8.16E-04 |
| **[18, 24)** | **[0.4, 0.6)** | **[0, 2)** | 3.05E-07 | 3.37E-04 | 3.75E-02 | 7.70E-01 | 1.92E-01 |
| **[18, 24)** | **[0.4, 0.6)** | **[2, 4)** | 1.81E-06 | 4.92E-04 | 1.12E-01 | 7.51E-01 | 1.37E-01 |
| **[18, 24)** | **[0.4, 0.6)** | **[4, 6)** | 2.07E-05 | 1.30E-03 | 4.28E-01 | 5.55E-01 | 1.55E-02 |
| **[18, 24)** | **[0.4, 0.6)** | **[6, 8)** | 2.22E-05 | 1.34E-03 | 4.41E-01 | 5.44E-01 | 1.40E-02 |
| **[18, 24)** | **[0.4, 0.6)** | **[8, 10]** | 2.22E-05 | 1.34E-03 | 4.41E-01 | 5.44E-01 | 1.40E-02 |
| **[18, 24)** | **[0.6, 0.8)** | **[0, 2)** | 6.64E-08 | 1.25E-04 | 6.82E-03 | 6.21E-01 | 3.72E-01 |
| **[18, 24)** | **[0.6, 0.8)** | **[2, 4)** | 1.38E-07 | 2.28E-04 | 2.83E-02 | 6.84E-01 | 2.88E-01 |
| **[18, 24)** | **[0.6, 0.8)** | **[4, 6)** | 1.19E-06 | 6.22E-04 | 1.75E-01 | 7.47E-01 | 7.80E-02 |
| **[18, 24)** | **[0.6, 0.8)** | **[6, 8)** | 1.26E-06 | 6.37E-04 | 1.84E-01 | 7.42E-01 | 7.35E-02 |
| **[18, 24)** | **[0.6, 0.8)** | **[8, 10]** | 1.26E-06 | 6.37E-04 | 1.84E-01 | 7.42E-01 | 7.35E-02 |
| **[18, 24)** | **[0.8, 1]** | **[0, 2)** | 2.37E-08 | 3.95E-05 | 2.89E-03 | 4.85E-01 | 5.12E-01 |
| **[18, 24)** | **[0.8, 1]** | **[2, 4)** | 6.59E-08 | 1.14E-04 | 9.36E-03 | 5.85E-01 | 4.05E-01 |
| **[18, 24)** | **[0.8, 1]** | **[4, 6)** | 3.16E-07 | 4.41E-04 | 6.13E-02 | 7.98E-01 | 1.40E-01 |
| **[18, 24)** | **[0.8, 1]** | **[6, 8)** | 3.26E-07 | 4.55E-04 | 6.48E-02 | 8.01E-01 | 1.34E-01 |
| **[18, 24)** | **[0.8, 1]** | **[8, 10]** | 3.26E-07 | 4.55E-04 | 6.48E-02 | 8.01E-01 | 1.34E-01 |
| **[24, 30]** | **[0, 0.2)** | **[0, 2)** | 1.88E-05 | 1.52E-03 | 3.82E-01 | 5.95E-01 | 2.17E-02 |
| **[24, 30]** | **[0, 0.2)** | **[2, 4)** | 6.95E-05 | 4.32E-03 | 4.89E-01 | 4.94E-01 | 1.25E-02 |
| **[24, 30]** | **[0, 0.2)** | **[4, 6)** | 2.94E-04 | 3.29E-02 | 7.60E-01 | 2.06E-01 | 1.34E-04 |
| **[24, 30]** | **[0, 0.2)** | **[6, 8)** | 3.04E-04 | 3.50E-02 | 7.66E-01 | 1.99E-01 | 1.31E-04 |
| **[24, 30]** | **[0, 0.2)** | **[8, 10]** | 3.04E-04 | 3.50E-02 | 7.66E-01 | 1.99E-01 | 1.31E-04 |
| **[24, 30]** | **[0.2, 0.4)** | **[0, 2)** | 1.41E-06 | 6.13E-04 | 1.70E-01 | 7.43E-01 | 8.66E-02 |
| **[24, 30]** | **[0.2, 0.4)** | **[2, 4)** | 1.52E-05 | 1.01E-03 | 2.80E-01 | 6.65E-01 | 5.44E-02 |
| **[24, 30]** | **[0.2, 0.4)** | **[4, 6)** | 1.19E-04 | 4.05E-03 | 6.23E-01 | 3.72E-01 | 1.08E-03 |
| **[24, 30]** | **[0.2, 0.4)** | **[6, 8)** | 1.26E-04 | 4.24E-03 | 6.32E-01 | 3.62E-01 | 9.80E-04 |
| **[24, 30]** | **[0.2, 0.4)** | **[8, 10]** | 1.26E-04 | 4.24E-03 | 6.32E-01 | 3.62E-01 | 9.80E-04 |
| **[24, 30]** | **[0.4, 0.6)** | **[0, 2)** | 2.70E-07 | 2.97E-04 | 3.31E-02 | 7.41E-01 | 2.26E-01 |
| **[24, 30]** | **[0.4, 0.6)** | **[2, 4)** | 1.60E-06 | 4.31E-04 | 9.98E-02 | 7.38E-01 | 1.62E-01 |
| **[24, 30]** | **[0.4, 0.6)** | **[4, 6)** | 1.83E-05 | 1.11E-03 | 3.88E-01 | 5.93E-01 | 1.86E-02 |
| **[24, 30]** | **[0.4, 0.6)** | **[6, 8)** | 1.96E-05 | 1.15E-03 | 4.00E-01 | 5.83E-01 | 1.67E-02 |
| **[24, 30]** | **[0.4, 0.6)** | **[8, 10]** | 1.96E-05 | 1.15E-03 | 4.00E-01 | 5.83E-01 | 1.67E-02 |
| **[24, 30]** | **[0.6, 0.8)** | **[0, 2)** | 5.86E-08 | 1.10E-04 | 5.85E-03 | 5.77E-01 | 4.17E-01 |
| **[24, 30]** | **[0.6, 0.8)** | **[2, 4)** | 1.22E-07 | 2.01E-04 | 2.50E-02 | 6.48E-01 | 3.27E-01 |
| **[24, 30]** | **[0.6, 0.8)** | **[4, 6)** | 1.05E-06 | 5.48E-04 | 1.56E-01 | 7.50E-01 | 9.35E-02 |
| **[24, 30]** | **[0.6, 0.8)** | **[6, 8)** | 1.11E-06 | 5.61E-04 | 1.64E-01 | 7.47E-01 | 8.81E-02 |
| **[24, 30]** | **[0.6, 0.8)** | **[8, 10]** | 1.11E-06 | 5.61E-04 | 1.64E-01 | 7.47E-01 | 8.81E-02 |
| **[24, 30]** | **[0.8, 1]** | **[0, 2)** | 2.09E-08 | 3.48E-05 | 2.49E-03 | 4.42E-01 | 5.56E-01 |
| **[24, 30]** | **[0.8, 1]** | **[2, 4)** | 5.82E-08 | 1.00E-04 | 8.17E-03 | 5.42E-01 | 4.50E-01 |
| **[24, 30]** | **[0.8, 1]** | **[4, 6)** | 2.79E-07 | 3.89E-04 | 5.44E-02 | 7.78E-01 | 1.68E-01 |
| **[24, 30]** | **[0.8, 1]** | **[6, 8)** | 2.88E-07 | 4.01E-04 | 5.75E-02 | 7.81E-01 | 1.61E-01 |
| **[24, 30]** | **[0.8, 1]** | **[8, 10]** | 2.88E-07 | 4.01E-04 | 5.75E-02 | 7.81E-01 | 1.61E-01 |

**(a)**


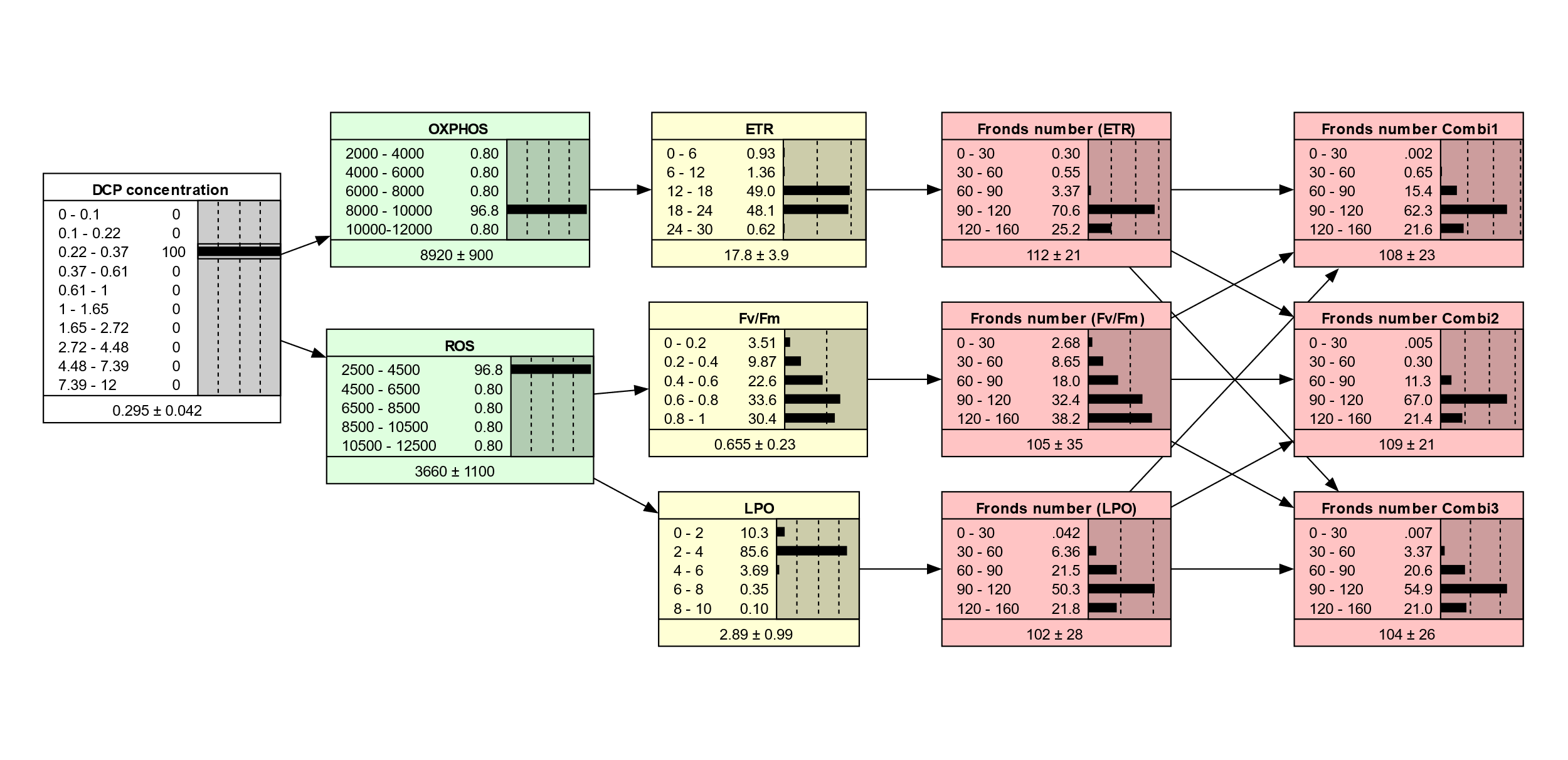


**(b)**


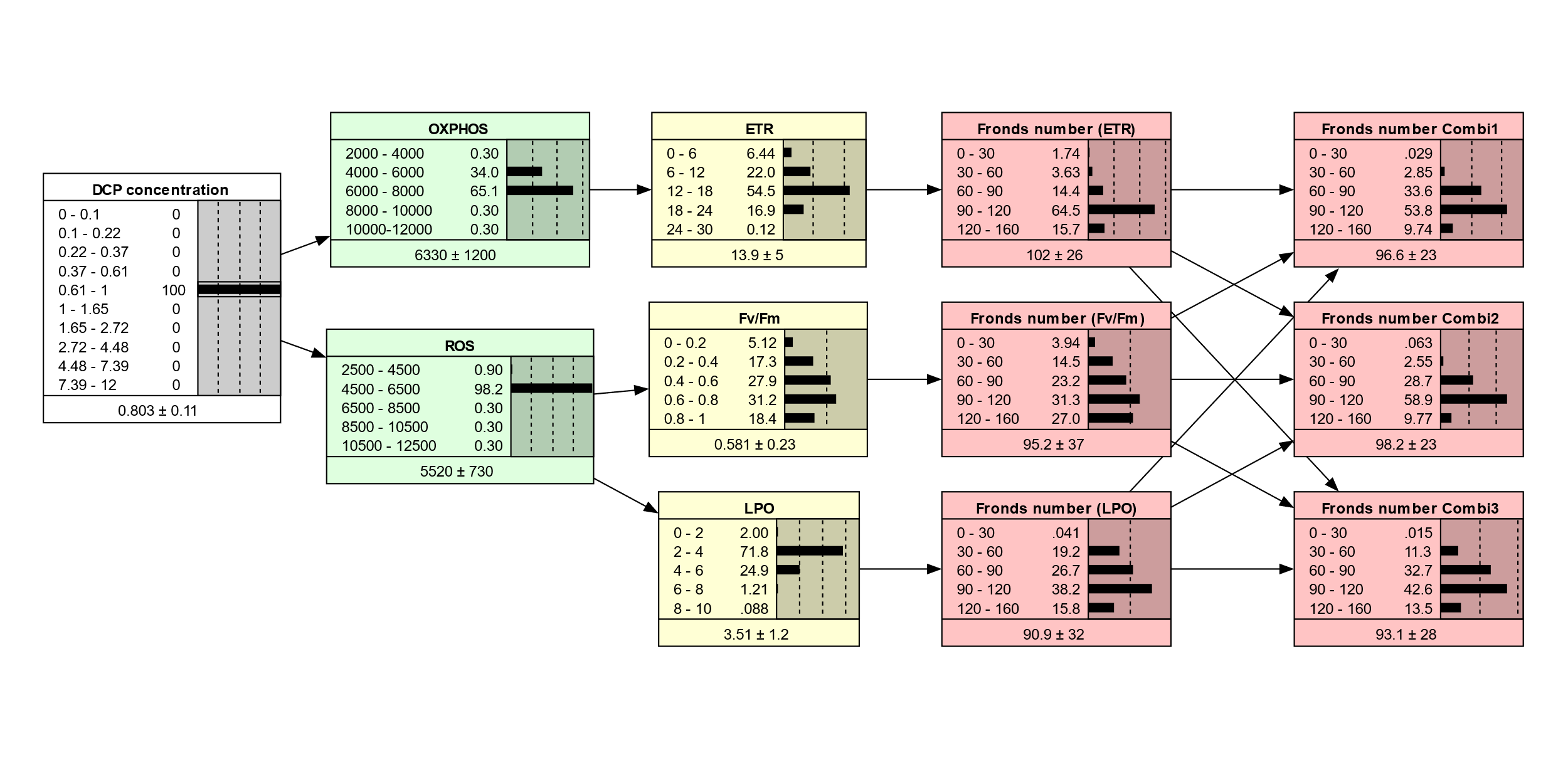


**(c)**


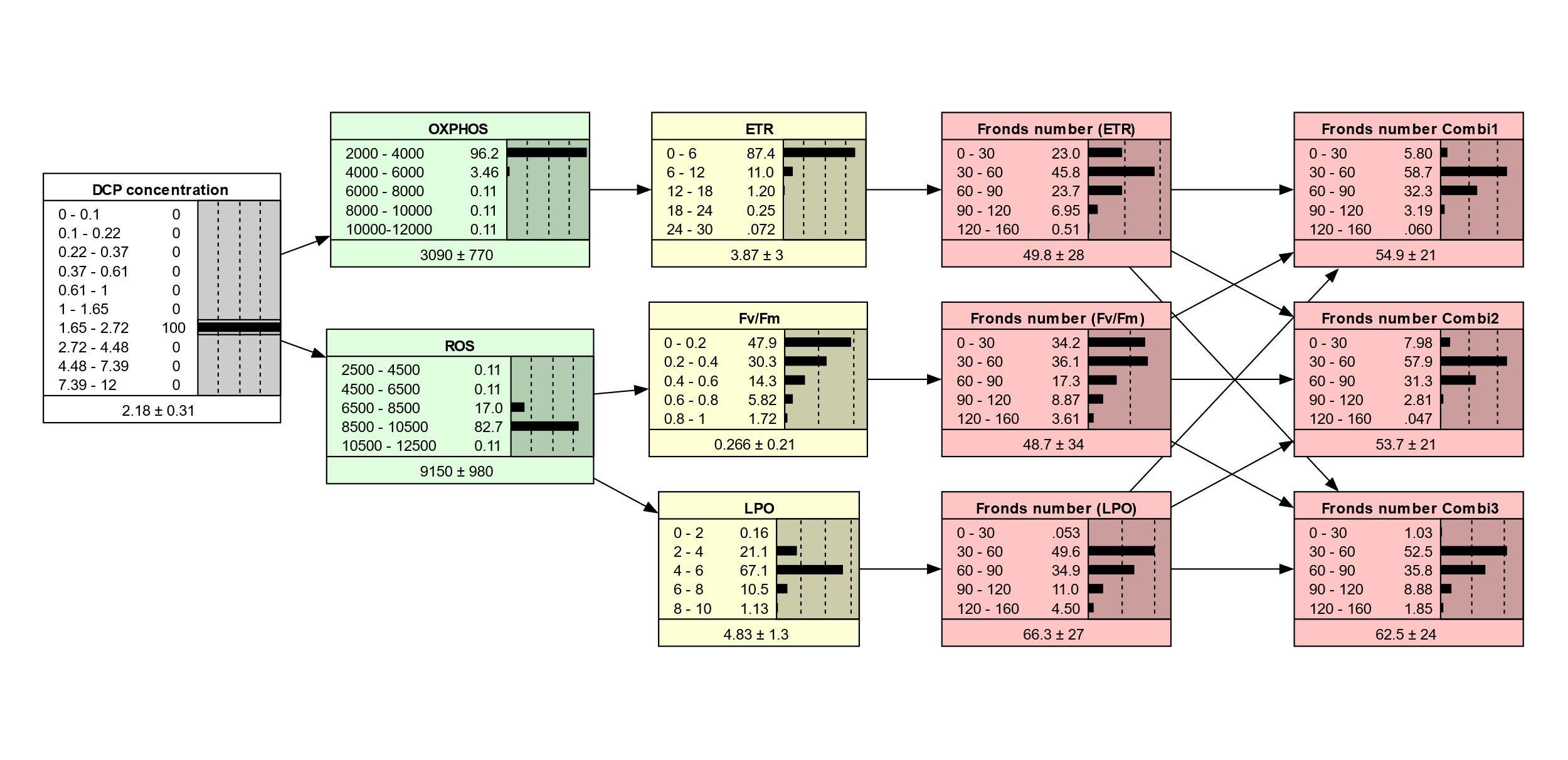


**Figure S3.** Adverse outcome predictions by the AOP-BN with the four alternative combination rules (Table 3). For combination rule 4, the predicted distribution of fronds number is identical to the node "Fronds number (ETR)".The three plots show the same scenarios (DCP concentrations) as in Figure 4. The final version of the AOP-BN model with combination rule 2 (Figure 4) is obtained by deleting the nodes "Fronds number Combi1" and "Fronds number Combi3", and absorbing the intermediate nodes "Fronds number (ETR)", "Fronds number (Fv/Fm)" and "Fronds number (LPO)".


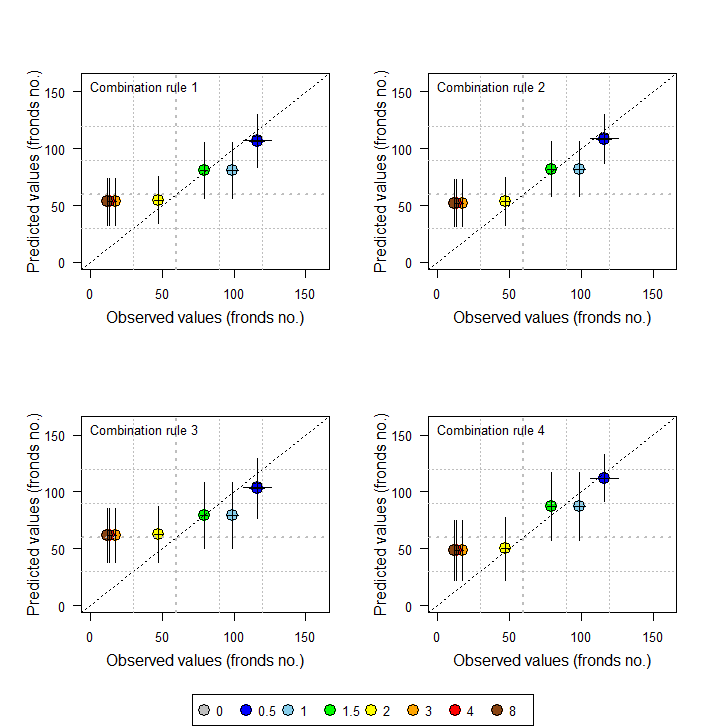


**Figure S4**. Internal validation of the AOP-BN by comparison of observed and expected mean values, for the model run from the stressor node with the four alternative combination rules. The colour code represents the experimental treatment (concentration of DCP in mg/L). Note that the response values for the two lowest concentrations cannot be discerned. The grid represents the intervals of the AO node; the thicker line indicates the 2-state intervals used for evaluation of accuracy (Table 5).
